# Decoding Rho GTPase signalling networks in directed cell migration

**DOI:** 10.3389/fcell.2026.1818051

**Published:** 2026-04-28

**Authors:** Yasaswi Gayatri Mishra, A. Indumathi, Saratchandra Singh Khumukcham, Bramanandam Manavathi

**Affiliations:** 1 Department of Biochemistry, School of Life Sciences, University of Hyderabad, Hyderabad, India; 2 Division of Gastroenterology, Department of Medicine, Duke University, Durham, NC, United States

**Keywords:** Cdc42, cell invasion, cell migration, Rac, Rho GTPases, RhoA, collective cell migration

## Abstract

Small G proteins are molecular switches in the realm of cell biology, switching from the GTP-bound active form to the GDP-bound inactive form. They participate in controlling various essential cellular functions, one of which is cell migration. Cell migration is a sine *qua non* for the proper functioning of the cell, as it aids in a litany of cellular processes, including embryonic development, morphogenesis, organogenesis, wound healing, and pathogenic states such as cancer metastasis. Amongst the five families of small G proteins (with over 150 members), Rho GTPase family is known to have significant roles in cell migration. Rho GTPases (primarily RhoA, Rac1, and Cdc42) translate extracellular cues into the spatiotemporal coordination of the actin cytoskeleton, allowing cells to polarize, migrate and navigate their environment. They integrate biochemical and biophysical signals through guanine nucleotide exchange factors (GEFs) and GTPase-activating proteins (GAPs). Rac1 and Cdc42 are known to act at the front end of a migrating cell and aid in cell polarisation, formation of protrusions and adhesive structures, while RhoA, though having overlapping roles, majorly functions at the cell rear, where it regulates cell body contraction and tail detachment, all of which we look into elaborately in this review. We also discuss the roles played by the other Rho GTPases in cell migration and have touched on the physiological and pathological impacts that Rho GTPase-regulated cell migration has on human health.

## 1 Introduction

### 1.1 Small GTPases

The Ras superfamily of small GTPases consists of more than 150 members categorized into five primary families Ras, Rho (Ras homology), Rab (Ras-like in brain), Arf (ADP-ribosylation factor) and Ran (Ras-like nuclear). First identified in the early 1980s through the discovery of Ras proto-oncogenes, these small G-proteins function as ubiquitous molecular switches that regulate a wide array of essential cellular processes (Kahn et al., 1992). Subsequently, Rad, Rap, Rheb and Miro were also added to the Ras superfamily (Song et al., 2019). Small GTPases function as binary molecular switches, transitioning between an inactive, GDP-bound state and an active, GTP-bound state. This cycle is strictly regulated by GEFs (guanine nucleotide exchange factors), which facilitate activation, GAPs (GTPase-activating proteins), which mediate inactivation, and GDIs (guanine-nucleotide dissociation inhibitors), which sequester the inactive protein in the cytosol. The high intracellular ratio of GTP to GDP provides the thermodynamic drive for activation, with the transition kinetically controlled by GEFs, which mediate the exchange of GDP for GTP to flip the molecular switch to the “on” state. Once signalling is complete, GAPs accelerate the intrinsic hydrolytic activity of the G protein, converting the bound GTP back to GDP to “off” the signal. To prevent premature reactivation, GDIs then sequester the inactive, GDP-bound protein in the cytosol (Cherfils and Zeghouf, 2013; Bourne et al., 1990). The small GTPases are crucial regulatory proteins conserved from yeast to mammals, involved in the regulation of a variety of functions in a eukaryotic cell. The Ras subfamily is known to have roles in mediating immunity, inflammatory responses, proliferation and differentiation. The Rab and Arf subfamilies play roles in vesicle trafficking and protein transport. In contrast, the Ran subfamily is involved in the cell cycle, cellular redox reactions, and the cell spindle apparatus, among other processes. The Rho subfamily, which we are primarily interested in, regulates various aspects of cell migration, including cell polarisation, plasma membrane (PM) tension, adhesion, and microtubule dynamics, as well as migratory structures of the cell (Song et al., 2019; Reiner and Lundquist, 2018). Rho GTPase subfamily comprised of 20 genes that can be further divided into 8 different subfamilies, *viz.*, (a) the Rho subfamily (RhoA, RhoB, RhoC), (b) the Rac subfamily (Rac1, Rac2, Rac3, RhoG), (c) the Cdc42 subfamily (Cdc42, RhoQ, RhoJ), (d) the Rnd subfamily (Rnd1, Rnd2, Rnd3), (e) the Rho BTB subfamily (Rho BTB1 and Rho BTB2), (f) RhoH subfamily, (g) RhoF/RhoD subfamily, and (h) RhoU/RhoV subfamily (Haga and Ridley, 2016). Of these, RhoA, Rac1 and Cdc42 are the most well characterised and have significant roles in cell migration, which we will be majorly looking into in this review.

### 1.2 Cell migration

Cell migration refers to the movement of cells from one location to another in response to various physiological and pathological cues. It forms a fundamental attribute of a cell in response to the proper functioning, development, and organization of a multicellular being. It is a highly complex, multi-step process that involves multiple signalling pathways, including those controlled by Rho GTPases, integrins, growth factors, and cytokines (Zegers and Friedl, 2014). In recent years, the molecular mechanisms underlying cell migration have been the focus of intense research; it plays a critical role in numerous physiological and pathological events, including embryonic development, organogenesis, maintenance of homeostasis, tissue repair, arthritis, multiple sclerosis and cancer metastasis. Cellular migration is typically characterized in 1D, 2D, or 3D contexts. While 2D and 3D migration both rely on integrin adhesion complexes (IACs) including focal adhesions, podosomes, invadopodia and hemidesmosomes as well as protrusive structures like lamellipodia and filopodia (Mishra and Manavathi, 2021). 3D environments introduce greater mechanistic diversity. Within the complex matrices, cells utilize specialized migration modes, including mesenchymal, amoeboid, lobopodial, and collective migration, to navigate the architectural constraints of the extracellular matrix (ECM) (Yamada and Sixt, 2019). All these processes are orchestrated by complex signalling pathways, which involve the coordination of multiple signalling inputs. One such input is the Rho family of GTPases, which act as molecular switches regulating various aspects of cell migration, including cytoskeletal rearrangements, adhesion dynamics, and polarisation (Ridley, 2015). The activity of Rho GTPases is tightly regulated by multiple upstream signalling inputs, including receptor tyrosine kinases, G protein-coupled receptors (GPCRs), and integrins. These inputs converge to modulate the activity of Rho GTPase regulators, the GEFs and the GAPs (Table 1, 2). A detailed review by Lawson and Ridley discusses the roles of GEFs and GAPs in forming regulatory complexes that spatially and temporally modulate Rho GTPase activity in cell migration and invasion (Lawson and Ridley, 2018). The convergence of multiple signalling inputs on Rho GTPase regulators provides a mechanism for integrating diverse extracellular signals to achieve coordinated regulation of Rho GTPase activity and downstream signalling (Schiller, 2006; Omble and Kulkarni, 2022). This integration is crucial for proper cell migration, as dysregulation of Rho GTPase activity can lead to defects in migration and adhesion, contributing to disease pathogenesis. This review will examine the significance of various inputs in regulating Rho GTPase activity and its downstream signalling pathways during cell migration, and discuss the implications of dysregulated Rho GTPase signalling in various diseases.

**TABLE 1:** List of Rho GTPase GAPs and their roles in cell migration. We have included only the most recent GAPs as the others have been listed in another review in details (Lawson and Ridley, 2018).

Sl. No.	GTPase	GAP	Effects	References
1	Cdc42	StarD13	Invasion and matrix degradation in prostate cancer and lung cancer	Jaafar et al. (2021), Al Haddad et al. (2020)
2	​	GRAF1	Migration and invasion of colorectal cancer cells	Xu et al. (2023)
3	​	ARHGAP44	Cell spreading and migration in H1299 (a lung carcinoma cell line)	Xu et al. (2017a)
4	​	ARHGAP21	Cell polarisation disruption in A549 (lung adenocarcinoma cell line)	Lee et al. (2018)
5	​	IQGAP1	Cell migration in HEK293 cells	Gorisse et al. (2020)
6	RhoA	StarD13	Cell migration in lung cancer	Al Haddad et al. (2020)
7	​	GRAF1	Partial role in enhancement of focal adhesions and stress fibers, cell elongation	Regev et al. (2017)
8	​	ARHGAP29	Cell migration in keratinocytes	Reeb et al. (2023)
9	​	ARHGAP4	2D migration, disordered 3D morphogenesis in HEK293 cell line	Kang et al. (2021)
10	​	ARHGAP11A	Cell spreading in breast cancer	Lawson et al. (2016)
11	​	p190A	Partial role in cell migration in endometrial cancer cells	Wen et al. (2020)
12	​	DLC1	Cell migration in human cancer cell lines	Wang et al. (2020b)
13	​	ARAP3	Cell invasion and metastasis in breast cancer	Zhang et al. (2023)
14	​	BPGAP1 (ARHGAP8)	Involved in polarized cell motility, spreading, invadopodium formation, cell extravasation and cancer cell migration	Wong et al. (2023)
15	​	Myo9b	Directional cell migration in macrophages	Sa et al. (2021)
16	Rho1	HUM7	Epidermal cell migration in embryonic morphogenesis in *Caenorhabditis elegans*	Wallace et al. (2018)
17	Rac	FilGAP	Regulates front-rear polarity and tumour migration through ECM in breast cancer cells	Saito et al. (2021)
18	​	​	Cell invasion in breast cancer	Tsutsumi et al. (2020)
19	​	RacGAP1	Cell spreading in breast cancer	Lawson et al. (2016)
20	​	ArhGAP45	Cell migration in human umbilical vein endothelial cells	Amado-Azevedo et al. (2018)
21	​	BCR	Cell migration in HEK293 cells	Borza et al. (2022)
22	​	ARHGAP22	Lamella formation and spreading of melanoma cells	Mori et al. (2022)
23	​	ARHGAP31	Podocytes cell migration and adhesion	Matsuda et al. (2022)
24	​	SRGAP1	Podocytes Lamellipodial Extension and Spreading	Rogg et al. (2021)

**TABLE 2:** List of Rho GTPase GEFs and their roles in cell migration. We have included only the most recent GEFs as the others have been listed in another review in details (Lawson and Ridley, 2018).

Sl. No.	GTPase	GEF	Effects	References
1	Cdc42	PDZ	Formation of filopodia like protrusions in HEK293T cells	Castillo-Kauil et al. (2020)
2	​	Asef4	promotes aberrant migration and invasion of colorectal cancer cells	Yang et al. (2021)
3	​	ARHGEF16	Induced colon cancer cell migration	Yu et al. (2020)
4	​	DOCK6	Positively correlates with lymph node metastasis, depth of invasion and vascular invasion in gastric cancer	Chi et al. (2020)
5	​	FGD4	increased cell migration, altered expression of EMT markers in PC3 cells	Bossan et al. (2018)
6	​	DOCK8	Induces T cell migration	Xu et al. (2017b)
7	​	ARHGEF15	Promotes migration and invasion of pancreatic cancer cells	Fukushima et al. (2016)
8	RhoA	VAV3	Oligodendrocyte precursor cell migration	Schäfer et al. (2022)
9	​	GEF-H1	FA turnover, controls leading edge dynamics by increasing RhoA activity at the leading edge and decreases cell migration	Smart et al. (2022)
10	​	​	Decrease in cell invasion by increase in RhoA activity and E-cadherin localisation to membranes in breast cancer	Kalpana et al. (2021)
11	​	ARHGEF3	Actomyosin contraction	(Zaritsky et al., 2017)
12	​	LARG	Regulation of cell migration, polarisation and adhesion	Ghanem et al. (2022)
13	​	PDZ	Involved in cell contraction in HEK293T cells	Castillo-Kauil et al. (2020)
14	​	Ect2	Involved in cortical actin nucleation via mDia1 in MEFs (mouse embryonic fibroblasts)	Limzerwala et al. (2020)
16	Rac1	VAV3	Oligodendrocyte precursor cell migration	Schäfer et al. (2022)
17	​	TRIO	Neural crest cell migration, controls protrusion formation	Kratzer et al. (2020)
18	​	DOCK1	Cytoskeleton rearrangement, actin polymerisation	Koubek and Santy (2022)
19	​	DOCK6	Positively correlates with lymph node metastasis, depth of invasion and vascular invasion in gastric cancer	Chi et al. (2020)
20	​	SOS1	Actin remodelling and migration in chronic myelogenous leukemic cells	Gerboth et al. (2018)
21	​	ARHGEF16	Induced colon cancer cell migration	Yu et al. (2020)
22	​	PREX1	Increased melanoma cell invasion	Ryan et al. (2016)
23	​	​	Drives lung fibroblast migration in pulmonary fibrosis	Liang et al. (2021b)
24	​	FARP1	Mediate lung cancer cell migration upon EGFR and c-Met activation	Cooke et al. (2021)
		ARHGEF39		
		TIAM2		

### 1.3 Importance of converging inputs for regulating Rho GTPase activity and downstream signalling pathways

**FIGURE 1:**
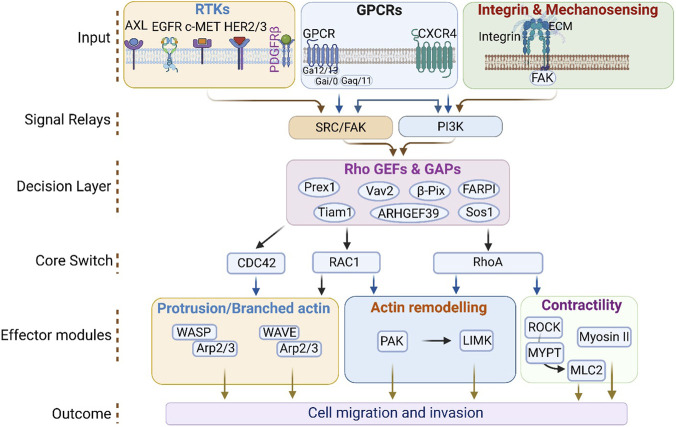
Hierarchical organization of receptor inputs to Rho GTPase signalling modules that drive cell migration and invasion. Extracellular cues sensed by receptor tyrosine kinases (RTKs; EGFR, HER2/3, c-MET, AXL), G-protein–coupled receptors (GPCRs), and integrins/mechanosensors (ECM engagement) converge on shared proximal hubs, prominently SRC/FAK and PI3K–PIP3. These hubs feed a limited set of Rho-family regulatory proteins (GEFs/GAPs)—illustrated by PREX1, SOS1, VAV2, TIAM1, β-PIX (ARHGEF39), and FARPI—that control the activation state of the core Rho GTPases RAC1, CDC42, and RhoA. Activated GTPases engage conserved effector modules that coordinate cytoskeletal remodeling: RAC1/CDC42 promote protrusion through WAVE/WASP–Arp2/3–dependent branched actin assembly and regulate actin dynamics via PAK–LIMK, whereas RhoA drives actin remodeling via PAK–LIMK and actomyosin contractility through ROCK–MYPT–MLC2 and myosin II. Collectively, integration across these tiers establishes the cytoskeletal and polarity states required for cell migration and invasion.

Rho family GTPases, particularly RhoA, Rac1, and Cdc42, function as central integrators of migratory signalling by translating a wide range of extracellular cues into coordinated changes in cytoskeletal organization, adhesion dynamics, and contractility. Their activation is not controlled by isolated receptor pathways, but rather by converging inputs from multiple receptor classes, including receptor tyrosine kinases (RTKs), GPCRs, integrins, and mechanosensory pathways. These diverse upstream signals are funnelled through a relatively limited set of intracellular relay modules, most prominently PI3K, SRC/FAK, and associated kinase networks which then regulate Rho GTPase activity primarily at the level of RhoGEFs and RhoGAPs. This hierarchical organization enables cells to integrate biochemical and biophysical information and convert it into precise, context-dependent migratory outputs (Figure 1).

Among RTK inputs, EGFR provides one of the best-characterized examples of convergence onto Rac1 and Cdc42 signalling. EGF stimulation activates multiple downstream relay pathways, including PI3K–AKT, Ras–MAPK, and Src-family kinases, which in turn engage Rac1 and Cdc42 through specific GEFs such as VAV2 (Omble and Kulkarni, 2022; Cotton and Claing, 2009; Pandey et al., 2000; Liu and Burridge, 2000). Rac1 is required for EGF-stimulated wound closure in intestinal epithelial cells through PI3K and Src-dependent mechanisms, whereas more recent work has shown that EGF-driven chemotactic motility depends on Rac1–VAV2 signalling (Pinilla-Macua et al., 2025; Dise et al., 2008). EGF can also stimulate NADPH oxidase via the PI3K/Rac pathway, resulting in elevated ROS production and increased migration (Sundaresan et al., 1996; Binker et al., 2009). In breast cancer cells, this Rac1-dependent ROS production further reinforces motility through PI3K/AKT/PAK1 signalling (Yang et al., 2011). Together, these observations place Rac1 at the centre of a convergence module in which RTK signalling, redox biology, and cytoskeletal remodelling are closely coupled.

An important feature of this RTK-driven Rac1 program is its capacity for crosstalk with adhesion pathways. Rac1 participates in EGFR–integrin signalling crosstalk through adaptor proteins such as p66Shc and Grb2, which increase Rac1-dependent ROS production. Under oxidative stress, p66Shc promotes a switch in SOS1 complex organization from the Grb2/SOS1 pool to the Eps8/E3b1 pool, thereby enhancing Rac1 activation and oxidant generation (Innocenti et al., 2002). Similarly, laminin-dependent signalling in skeletal muscle activates a dystroglycan–syntrophin–Grb2–SOS1–Rac1–PAK1–JNK axis, further illustrating how adhesion-associated cues feed into Rac1-centered signalling networks (Oak et al., 2003). These examples underscore that convergence is not simply a matter of multiple receptors activating the same GTPase, but rather that upstream signals are reinterpreted through adaptor complexes and scaffold dynamics to shape the quality of Rac1 output.

The centrality of the GEF/GAP layer is further emphasized by the variety of RacGEFs that operate downstream of convergent RTK and GPCR inputs. EGFR expression correlates positively with TIAM1, and EGFR can activate a PI3K/AKT/TIAM1/Rac1 signalling axis that enhances tumour progression (Yang et al., 2011). Additional RacGEFs, including FARP1, ARHGEF39, and TIAM2, function downstream of EGFR, c-MET, and AXL receptor tyrosine kinases and promote motility through an AXL–Gab1–PI3K pathway (Cooke et al., 2021). Likewise, P-Rex1 integrates upstream signals from HER/ErbB receptors and the GPCR CXCR4, providing a direct molecular mechanism by which RTK and GPCR pathways converge on Rac1 (Kazanietz et al., 2018). VAV2 itself is activated downstream of both EGF and PDGF, highlighting how multiple ligand–receptor systems are integrated at the level of shared GEFs (Pandey et al., 2000; Liu and Burridge, 2000). Thus, the regulatory layer composed of GEFs and GAPs acts as the critical node at which receptor diversity is translated into specific Rho GTPase outputs.

Cdc42 is incorporated into this convergent signalling architecture in a related but functionally distinct manner. In response to EGF stimulation, Cdc42 is activated through PI3K, Src, and VAV2, and in some contexts its activation precedes that of Rac1 (El-Sibai et al., 2007; Maldonado et al., 2020). Cdc42 is particularly important for organizing adhesive structures behind the leading edge and for regulating the formation of protrusive actin-based structures. Scaffold proteins further sharpen this spatial specificity; for example, IQGAP1 forms a complex with PIPKIγ, PI3K, talin, and Cdc42 following EGF stimulation, thereby linking phosphoinositide signalling, polarity scaffolds, and actin regulation to directed migration (Yerramilli et al., 2022). Notably, both Rac1 and RhoA can also activate PIPKIγ, reinforcing the idea that shared regulatory intermediates connect otherwise distinct branches of the Rho GTPase network (Weernink et al., 2004).

In contrast to the predominantly protrusive roles of Rac1 and Cdc42, RhoA is positioned as the principal regulator of actomyosin contractility and mechanical adaptation. RhoA activation is strongly linked to GPCR signalling, particularly through extracellular lipids such as lysophosphatidic acid (LPA) and sphingosine-1-phosphate (S1P), which signal via Gα12/13 to recruit Rho-specific GEFs such as p115RhoGEF and LARG (Xiang et al., 2013). However, RhoA is not restricted to GPCR signalling alone. Growth factors such as EGF and TGF-β also provide spatiotemporal signals that activate RhoA, thereby establishing the relay between Rac1-driven protrusion at the front of the cell and RhoA-mediated actomyosin contraction required for forward translocation (Bhowmick et al., 2001; Tong et al., 2016). Consistent with this, EGF stimulation promotes aPKCζ-dependent recruitment of RhoA to the plasma membrane, leading to phosphorylation of myosin light chain and inhibition of myosin phosphatase, both of which are essential for directed migration and adhesion dynamics (Petrov et al., 2017). Moreover, EGFR can activate the RhoA/ROCK1/LIMK2/cofilin signalling cascade to promote motility and invasion (Wang J. et al., 2018; Crosas-Molist et al., 2022).

RhoA acts as an important mechanosensitive regulator, converting extracellular physical cues into intracellular biochemical responses. Matrix viscosity, stiffness, and fluid shear stress all influence RhoA-dependent signalling. Increased matrix viscosity elevates RhoA-mediated contraction through an ARP2/3-dependent actin network, which increases NHE1 activity, membrane tension, and TRPV4-dependent Ca^2+^ influx, thereby promoting RhoA activation and migration under high-viscosity conditions (Bera et al., 2022). Fluid shear stress similarly activates RhoA-dependent programs. In liver cancer stem cells, shear stress promotes migration through RhoA-mediated YAP1 dephosphorylation and nuclear localization, whereas high shear stress activates TRPM7, resulting in RhoA/myosin II activation and migration reversal during intravasation. In this context, RhoA—but not Rac1 or Cdc42—acts as a fluid sensor mediating shear-induced migration (Yan et al., 2022; Yankaskas et al., 2021). The flow of lymphatic drainage in prostate cancer also enhances motility through RhoA/ROCK-dependent activation of YAP (Mohammadalipour et al., 2022). These findings support the view that mechanical inputs are not peripheral to Rho signalling, but instead converge directly onto the same decision-making architecture that processes biochemical receptor signals.

Matrix stiffness provides a further example of how biochemical and biophysical signalling are integrated through Rho GTPases. Increased stiffness of the ECM surrounding tumour cells is associated with elevated RhoA, Rac1, ROCK1/2, and MMP expression, as well as enhanced migration and invasive behaviour. Mechanistically, stiffness-dependent migration has been linked to RhoA–mDia-mediated microtubule detyrosination, β1-integrin/FAK activation, and focal adhesion maturation. In ovarian cancer, a stiffness-responsive Src/TAGLN axis promotes RhoA/ROCK-mediated invasion and proliferation, further illustrating how ECM mechanics are integrated into the Rho signalling network (Wang T. et al., 2017; Zhao et al., 2018; Peng et al., 2019; Wei et al., 2021). Thus, mechanosensory signalling should be viewed not as a separate layer of regulation, but as an integral component of the convergent Rho GTPase signalling network.

Although RhoA, Rac1, and Cdc42 have distinct roles, their downstream signalling pathways also display substantial functional convergence. Rac1/Cdc42-driven pathways such as PAK signalling and RhoA-driven pathways such as ROCK–LIMK both regulate actin dynamics, while MLC2-dependent mechanisms link multiple Rho GTPases to contractility. The result is that distinct upstream inputs and distinct GTPase branches ultimately converge on a relatively small set of shared effector modules controlling protrusion, actin remodelling, and contractile force generation. This convergence at both the input and effector levels explains how cells can coordinate leading-edge extension, adhesion turnover, and rear retraction in a temporally and spatially organized manner.

Overall, converging inputs from receptor signalling, cell–matrix adhesion, and mechanical stress are essential for regulating Rho GTPase activity and downstream signalling pathways. By integrating these multiple sources of information through common relay pathways and a central GEF/GAP decision layer, cells achieve the precise control over cytoskeletal dynamics and force generation required for efficient migration and invasion. In this sense, the importance of converging inputs lies not simply in the number of pathways involved, but in the ability of cells to transform diverse extracellular signals into a coherent and adaptable migratory program.

### 1.4 Methods to resolve Rho GTPase dynamics in space and time

Progress in understanding Rho GTPase signalling has depended not only on the identification of upstream regulators and downstream effectors, but also on the development of methods capable of visualizing and perturbing GTPase activity in living cells (Kim et al., 2019). Whereas early biochemical approaches established the conceptual framework of the field, modern imaging- and perturbation-based methods have revealed that RhoA, Rac1, and Cdc42 do not operate as simple binary switches. Instead, they function as dynamic, spatially organized, and mechanically integrated signalling modules whose coupling is strongly influenced by cellular context, including whether migration occurs in two-dimensional or three-dimensional environments (Valon et al., 2017; de Beco et al., 2018; Pertz, 2010).

The earliest studies of Rho GTPase activation relied largely on pull-down assays, in which effector-binding domains such as the Rhotekin RBD or the PAK CRIB motif were used to isolate the GTP-bound fraction of RhoA, Rac1, or Cdc42 from cell lysates (Ito et al., 2018; Benard and Bokoch, 2002). These assays were instrumental in defining stimulus-response relationships and identifying upstream signalling pathways, but their limitations are intrinsic (Burridge and Wennerberg, 2004). Because pull-down approaches provide static, population-averaged measurements, they cannot resolve front-rear polarity, subcellular activation zones, or rapid activation-inactivation cycles (Pertz, 2010; Fritz and Pertz, 2016). Consequently, they obscure the transient and oscillatory signalling dynamics that drive protrusion, retraction, and turning during migration (Martin et al., 2016).

A major conceptual shift came with the development of genetically encoded biosensors, particularly probes compatible with Förster Resonance Energy Transfer (FRET) and Fluorescence Lifetime Imaging Microscopy (FLIM), which enabled direct, real-time visualization of Rho GTPase activity in single living cells. These approaches showed that Rac1 and Cdc42 activity is enriched at protrusive regions, whereas RhoA activity is dynamically distributed across both protrusive and retractile compartments, thereby challenging earlier models that assigned mutually exclusive spatial functions to individual GTPases (El-Sibai et al., 2007; Pertz, 2010; Pertz et al., 2006). Improved second-generation biosensors further revealed that Rho GTPase signalling is highly dynamic, occurring in localized and pulsatile bursts that are coordinated across space and time (Martin et al., 2016; Machacek et al., 2009). These studies established that migratory behaviour emerges from coupled signalling cycles rather than from sustained global activation states.

High-resolution live-cell imaging extended this view by resolving temporal coordination between protrusive Rac1/Cdc42 activity and RhoA-associated retraction dynamics, thereby providing a mechanistic basis for protrusion-retraction coupling during migration (Martin et al., 2016; Nanda et al., 2023). More recent work combining biosensor imaging with acute perturbation showed that this coupling is mediated in part by specific RhoGEFs, including ARHGEF11 and ARHGEF12, which coordinate Rac-Rho crosstalk to regulate protrusion-retraction cycles and exploratory migration (Nanda et al., 2023). Together, these findings repositioned Rho GTPase signalling as a dynamic, self-organizing system rather than a simple linear pathway (Lawson and Ridley, 2018; Martin et al., 2016; Nanda et al., 2023).

Live-cell biosensor approaches have also expanded from single-molecule measurements to the simultaneous visualization of broader signalling networks. Notably, concurrent imaging of EGFR, Ras, Rac1, and Rab5 demonstrated that spatial bias in Rac1 and Rab5 activation can arise even when receptor activation itself remains uniform across the plasma membrane (Jikko et al., 2025). These observations indicate that front-rear polarity is not passively inherited from receptor activation patterns, but instead emerges through downstream network-level processing involving feedback and trafficking-dependent coupling. In collective migration, Rac1 thus functions as a central determinant of front-side specification by integrating receptor-derived signals with cytoskeletal and endocytic dynamics (Jikko et al., 2025; Li et al., 2020).

The extension of Rho GTPase imaging into three-dimensional matrices further emphasized the context dependence of GTPase regulation. FRET-based measurements in fibrillar collagen showed that signalling logic in 3D differs fundamentally from that observed on conventional 2D substrates (Kutys and Yamada, 2014). Using this approach, β-PIX and srGAP1 were identified as matrix-specific regulators that couple Cdc42 and RhoA signalling during 3D collagen migration, selectively suppressing RhoA downstream of Cdc42 and demonstrating that extracellular matrix architecture can reprogram the internal logic of the Rho GTPase network (Kutys and Yamada, 2014). These findings illustrate how adapting biosensor-based measurements to physiologically relevant 3D microenvironments can uncover regulatory relationships that remain hidden in standard 2D migration assays (Kutys and Yamada, 2014; Petrie and Yamada, 2016).

Beyond observation, optogenetic and chemogenetic approaches enabled direct tests of causality with subcellular precision. Local optogenetic activation of Rac1 was sufficient to induce protrusion, directional turning, and symmetry breaking, whereas localized activation of Cdc42 generated polarized signalling and directional migration (Wu et al., 2009; O’Neill et al., 2016). Optogenetic control of RhoA-dependent contractility further demonstrated that local mechanical outputs can be manipulated with comparable spatial and temporal precision, highlighting the importance of long-range coupling between protrusive and contractile modules (Valon et al., 2017). Gradient perturbation experiments additionally revealed functional specialization among GTPases, with Cdc42 exerting a dominant role in directional sensing and front organization, while Rac1 primarily amplified protrusion and migration speed (de Beco et al., 2018). Together, these studies established that the spatial patterning of Rho GTPase activity is not merely correlative, but functionally instructive (Valon et al., 2017; de Beco et al., 2018; Wu et al., 2009; O’Neill et al., 2016).

Collectively, these methodological advances have redefined how Rho GTPase signalling is conceptualized. Classical biochemical assays established whether a GTPase was activated, but modern biosensors and optogenetic approaches now reveal where activation occurs, when it is initiated, how long it persists, and how it is integrated with structural and mechanical cues. As a result, the field has moved from a static, pathway-centric framework toward a dynamic systems view in which RhoA, Rac1, and Cdc42 are understood as spatially coordinated, context-dependent regulators of cell migration across diverse microenvironments (Pertz, 2010; Fritz and Pertz, 2016; Garcin and Straube, 2019; Devreotes and Horwitz, 2015; Bement et al., 2024).

## 2 Role of Rho GTPases in different steps of cell migration

**FIGURE 2:**
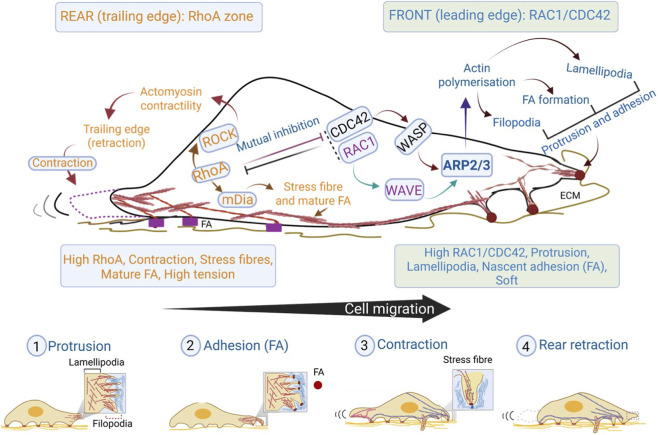
Spatial segregation and feedback control of Rho GTPase signalling during cell migration. A polarized migrating cell is organized into a leading-edge Rac1/Cdc42 zone and a rear RhoA zone. At the front, RAC1 activates WAVE–Arp2/3 to drive branched actin assembly and lamellipodial protrusion, while CDC42 activates WASP–Arp2/3 to promote filopodia formation and support front identity. Nascent focal adhesions (FAs) form within protrusions to couple actin polymerization to substrate engagement. Toward the rear, RhoA signals through ROCK to stimulate myosin II–dependent actomyosin contractility, promoting cell-body translocation and trailing-edge retraction; RhoA also supports stress fiber formation and FA maturation. Mutual antagonism between Rac1/Cdc42 and RhoA reinforces front–rear polarity by restricting protrusive signalling to the leading edge and contractile signalling to the trailing edge. A tension-dependent mechanochemical feedback between FA maturation and actomyosin force couples adhesion dynamics to contractility, stabilizing directional movement. The bottom schematic summarizes the migration cycle as four coordinated steps: (1) protrusion, (2) nascent adhesion formation, (3) contraction/translocation, and (4) rear retraction.

On a broad scale, cell migration and processes that prelude it are divided into six different steps, *viz.*, (a) Cell polarisation in response to various cues, (b) Formation of protrusive structures e.g., lamellipodium, filopodium, and invadopodium, (c) Formation of new adhesions at the leading edge, (d) matrix degradation (e) Contraction of cell body and tail detachment at the cell rear so that cell is displaced. Overall, Rac and Cdc42 act at the front of the cell, aiding in polarity, the formation of protrusive structures, and integrin-ECM engagement. RhoA acts at the rear end, aiding in actomyosin contractility (Figure 2). Here, we shall discuss the roles Rho GTPases play in each of the mentioned steps.

### 2.1 Role of Rho GTPases in cell polarisation

Cell polarity is the organization of the biochemical and cytoskeletal components of the cell that allows the cell to align along a geometric axis with defined ends, which is essential for several key processes, including directional cell growth, immune response, cell division, and cell migration. Polarity in cells can be induced both intrinsically and extrinsically via diffusible components forming a chemical gradient, i.e., biochemical signalling, which is different from mechanical cues that depend on the stiffness, shear, stress from the matrix, and also the elasticity and viscosity of membranes and the cytoskeleton (which was discussed above) (Turing, 1990; Goehring and Grill, 2013). The epithelial cells form an apical-basal polarity, where cells in one plane in a tissue exhibit planar polarity, and cells in migration exhibit front-to-back polarity, which we are interested in (Piroli et al., 2019). This front-to-back polarity formed under varying cues leads to directed cell migration in response to (a) diffused chemicals called chemotaxis, (b) chemicals on substrate called haptotaxis, (c) mechanical stiffness of substrate called durotaxis, (d) topographic nature, such as density of substrate called topotaxis and (e) electric fields called galvanotaxis (SenGupta et al., 2021; Park et al., 2018; Shellard and Mayor, 2020).

Cell polarity is fundamentally anchored by the Cdc42, which acts as the master molecular compass for directional organization. The initiation of polarity begins when Cdc42 clusters at a single point on the plasma membrane. A substantial amount of research is available on yeast cells that require Cdc42 to establish and reorient polarity in response to chemical gradients, as exemplified by pheromones (Ghose et al., 2022). Abnormally enhanced activity of Cdc42 via the PTEN/AX2/Cdc42 axis leads to a loss of cell polarity, increased migration and proliferation associated with pulmonary vascular remodelling in hepatopulmonary syndrome (Gao et al., 2019). Various mathematical models have been developed to describe cell polarisation and cell migration in 1D, 2D, and 3D, depicting the roles of RhoGTPases in the same context (Goehring and Grill, 2013; Buttenschön et al., 2020). In 2008, Mori et al., introduced a model for cell polarisation called the wave pinning model where they described how the cycling of active and inactive forms of Rho GTPases between the PM and cytoplasm could form a basis for the mechanism for cellular polarisation (Kopfer et al., 2020).

Once localized, Cdc42 recruits the Par3/Par6/aPKC scaffold (Goldstein and Macara, 2007). This complex physically organizes the cell’s interior, directing the Golgi apparatus and centrosome toward the front to facilitate the targeted transport of lipids and proteins needed for membrane expansion (Schmoranzer et al., 2009; Matsui et al., 2015). Reduction in active Cdc42 (Cdc42-GTP) as in congenital biliary atresia, the polarity complexes that are normally formed with Par6/Par3/αPKC complex were also reduced (Zhou et al., 2021). With respect to the fact that the Golgi apparatus becomes polarised towards the direction of migration, Cdc42 is present in the Golgi as well, and different pools of Cdc42 act together for a cell to form and maintain polarity (Farhan and Hsu, 2016). Active Cdc42 plays a crucial role in maintaining cell polarity by regulating microtubule and actin skeleton dynamics through an array of proteins, including WASP, PAK, Par6, IQGAP, and RalA, which are involved in multiple signalling pathways, as described by Sandrine Etienne-Manneville (Etienne-Manneville, 2004). It was found that angiopoietin-1 induced Cdc42 via PAK2/Paxillin and also Par3 (of Par polarity complex) and Cdc42 associates at the leading edge of migrating endothelial cells to induce cell polarity (Boscher et al., 2019). The interaction between polarity complexes and Rho-GTPases in a cell, both during collective and cell migration, with respect to normal and cancer cell migration, has been extensively reviewed by Gandalovičová et al. (2016).

Although the majority of the regulation of cell polarity is mediated by Cdc42, research has also found roles for Rac1 and RhoA. A review by Saha et al., highlights how cell-wide coordination emerges from a mechanochemical circuit, where physical forces specifically membrane tension function as global integrators that engage with self-organized actomyosin dynamics and biochemical signalling to orchestrate processes ranging from individual cell polarity to collective migration (Saha et al., 2018). mTORC2 was identified as a pivotal mechanosensitive integrator that recruits a PLD2-mTORC2 signalling axis to indirectly inhibit actin nucleation. This biochemical feedback loop is mathematically predicted to be superior to direct mechanical resistance in enabling competition between fronts and suppressing secondary pseudopods to maintain persistent polarity (Diz-Muñoz et al., 2016). Later mTORC2 was shown to bifurcate polarity control where its kinase-independent role gated the tension-based inhibition of leading-edge Rac/F-actin signals to prevent protrusion competition, while its kinase-dependent activity orchestrated RhoA-mediated trailing-edge contractility to ensure persistent migration under confinement (Saha et al., 2023). It was found that mice deficient in Rac1 in the second heart field, which are multipotent progenitor cells of the heart, experienced a loss of Scrib, an important protein involved in planar polarity and decreased expressions of WAVE and Arp2/3, leading to loss in polarity and migratory capability of cells, resulting in developmental defects in embryonic heart (Leung et al., 2014). Improper spatial activation of Rac1 by its GEF TRIO in the absence of centrosomes in a cell leads to increase in cell migration by rapid FA turnover and a loss of cell polarity (Cheng et al., 2019). In a literature review by Fukata et al., the regulation of cell polarisation via microtubules by Rac1, RhoA, and Cdc42 through modulation of specific effectors, such as mDia, IQGAP1, Par6, and PAK, has been described (Fukata et al., 2003). An interesting study by Lee H et al., observed that the cell motility is governed by co-ordinated Rho-GTPase effector ensemble which in two distinct modules produce opposite behavioural outputs. The Cdc42–FMNL interaction restricts Cdc42 activity to the cell front, establishing and reinforcing front-rear polarity, while the Rac1–ROCK interaction creates frontal contractility that generates arc stress fibres and enables spontaneous orthogonal turning (Lee et al., 2025).

In neural crests and gliomas, the relocation of αE-catenin bound to p115RhoGEF from lamellipodia to the rear leads to accumulation of active RhoA in the perinuclear region, where polarised Myosin IIB arcs are assembled for directed cell migration. Thus, catenins, via RhoA, regulate cell migration by stabilizing cell polarity (Vassilev et al., 2017). Rho family-interacting cell polarisation inhibitors are protein regulators that interact with RhoA, RhoB, and RhoC members to influence their downstream effects, which are involved in cell polarity and migration. Their detailed mechanism and role have been described in a short review by Lv et al. (2022). During alveolarization in lung development, the alveolar myofibroblasts migrate to septal tips, which is shown to be inhibited by lipopolysaccharides (LPS). Increased LPS upregulates RhoA activity via the TGF-α/EGFR/14-3-3β signalling pathway, which accounts for the disturbed polarisation and defective migration of myofibroblasts, leading to alveolarization arrest, a condition often seen in bronchopulmonary dysplasia (Li and Gong, 2020).

### 2.2 Role of Rho GTPases in formation of protrusive structures in the cell

Once the cell polarity is established Cdc42 drives the formation filopodia, which are spike-like protrusions enriched with actin filaments, serving as sensing devices for chemotropic cues and directing cellular movement (Wozniak et al., 2004). N-WASP-dependent actin polymerization downstream of Cdc42 causes filopodia to extend beyond the leading edge (Takenawa and Suetsugu, 2007). Various other studies have also shown the roles of Cdc42 in filopodia formation and the molecular mechanisms by which proteins like Rab6, Rab5a, ADP-ribosylation factor (Arf)-like 4A (Arl4A) and Robo1 that control it (Ma et al., 2020; Yuan and Wei, 2021; Vestre et al., 2019; Chiang et al., 2019). A CTRP8-RXFP1-JAK3-STAT3 axis was shown to promote filopodia formation in glioblastoma cells via Cdc42 (Glogowska et al., 2022). In an aggressive skin cancer, Merkel cell carcinoma, it was found that expression of the small T antigen by Merkel cell polyomavirus (MCPyV) led to filopodia formation by interacting with the catalytic subunit of protein phosphatase 4 (PP4C) leading to dephosphorylation of β1 integrin and was also was influenced by RhoA and Cdc42 activities (Stakaitytė et al., 2018). Mechanical stimuli like providing low shear stress to cells could also enhance Cdc42 dependent filopodia formation (Liu L. et al., 2019). An E3 ubiquitin ligase RNF8 was shown to mediate cell migration in triple negative breast cancer cells by regulating filopodia and FA formation via Cdc42 and RhoA (Pereira De Carvalho et al., 2021).

Upon signal detection through filopodia, Cdc42 recruits Rac1 GEFs like TIAM1, β-PIX, and DOCK to the site activating Rac1 (Table 2). Once active, the active Rac1 GTP then binds to the WAVE regulatory complex (WRC) activating it which in turn activates the Arp2/3 complex. This nucleates a dense, branched actin meshwork that physically pushes the membrane forward. This pushing of the membrane involves the formation of cellular protrusions called lamellipodia. The ways in which actin is involved in the formation of these protrusions are explained in detail in another review (Svitkina, 2018). The WRC is a multi-protein complex that consists of several protein subunits, including HSPC300, WAVE1/2 (wave homology domain), Abl interactor 1/2/3, NCK-associated protein 1, and specifically Rac1-associated protein 1. It plays a crucial role in promoting the assembly of actin filaments, which drives the protrusion of the leading edge of the cell during cell migration. WRC recruits to the PM through interactions with several proteins, including phosphatidylinositol-(3,4,5)-triphosphate and the 53 kDa insulin receptor substrate (IRSp53). The binding of GTP-bound Rac1, increases the affinity of WAVE2 for IRSp53, which promotes the ability of WAVE2 to stimulate Arp2/3 mediated actin polymerization (Rana et al., 2021). Rac1 immobilization at the cell edges depends on WRC binding, and this immobilization correlates with its activation (Mehidi et al., 2019). In addition, the binding of Rac1 to the D site of WRC enhances the binding of Arf-family GTPases, Arf1, to the surface of Sra1 in the WRC complex. The recruitment of Arf1 can directly activate WRC, regardless of Rac1 binding (Yang et al., 2022). This suggests that the WRC acts as a central actin regulator that integrates signalling from both Rac1 and Arf GTPases to promote Arp2/3-mediated actin polymerization and lamellipodia formation. Another Dbl family RhoGEF ARHGEF7/β-PIX and its binding partner, p21-activated kinase PAK1A (ARHGEF7-PAK1 complex) translocate in the lamellipodia protrusion. Further study shows that it is regulated by STIL and is crucial for the polarized formation of Rac1-mediated leading edge (Ito et al., 2020). A recent study shows that cell protrusions have high GTP levels, which causes Rac1 activation. Guanylate metabolism enzymes, including IMPDH2, enhance the GTP pool in cell protrusions, and their interaction with Rac1 promotes its activity and cell invasion (Bianchi-Smiraglia et al., 2021). Also, another study showed that receptor tyrosine kinase EPHA4 phosphorylates GMPR at Tyr267, decreasing GTP pools in cell protrusions and levels of GTP-bound Rac1, hence restricting cell invasion (Wolff et al., 2022). This showed that the activation of Rho GTPases, and therefore the invasive capabilities of the cells, are also dependent on the spatial regulation of GTP within the cell. Some proteins, such as CK1α, indirectly stabilize WAVE and contribute to membrane protrusion. CK1α interacts with WAVE and phosphorylates the acidic region within the VCA domain, promoting the stability of WAVE and protecting it from proteasomal degradation. This phosphorylation of the cutting region within this domain promotes its stability rather than its actin nucleation function (Hirschhäuser et al., 2021). Additionally, CYRI has been shown to interact directly with activated Rac1 and promote plasticity by opposing the recruitment of the active Scar/WAVE complex to leading edges during epithelial cystogenesis (Fort et al., 2018). However, n a 2022 study, it was found that the formation of lamellipodia-like structures could be independent of WRC. They found that upon WRC subunit disruption and treatment of cells with serum/growth factor stimulation/active GTPase expression, Arp2/3-dependent lamellipodia-like structures were formed, requiring both Rac and Cdc42, as well as other lamellipodial regulators, but were devoid of Ena/VASP (Kage et al., 2022). In a similar study by Schaks et al. it was found that the presence of Rac at the PM is not essential for the activation of WRC in lamellipodia formation; however, it required the presence of Rac GTPases. Surprisingly, they also found a redundancy of Rac GTPases as well in lamellipodia formation, provided some other mechanism previously activated WRC, and there was constitutive overexpression of either RhoG or Cdc42 (Schaks et al., 2021).

The interconversion of different protrusive structures and the roles of Rho GTPases in this process have been described in detail by Schaks et al. (Schaks et al., 2019). Migrating cells switch between lamellipodium-based and bleb-based migration modes. This requires a delicate balance between Rac1 and Rho/ROCK signalling. A switch-like mechanism was described by Ard et al., where they showed that phosphorylation of Diacylglycerol kinase ζ (DGKζ), which forms phosphatidic acid by phosphorylating diacylglycerol, by protein kinase Cα (PKCα) led to switching between Rac1 and RhoA signalling and catered to different modes of migration, *viz.*, lamellipodia or bleb-based migration (Ard et al., 2021). During lamellipodium formation, RhoA is essential for limiting the lamellipodium to a single part of the membrane, thereby facilitating cell motility. RhoA via ROCK activates myosin light chain 2 and MYPT to induce actomyosin contractility required for lamellipodium retraction rearwards. It prevents the formation of unproductive protrusions at the leading end. Thus, RhoA mediates spatial regulation of Rac activity (Vega et al., 2011). Various mechanisms explain the coordination of spatiotemporally separated Rho and Rac activity during cell migration. This is primarily achieved through the antagonistic action of RhoA on Rac1 and *vice versa*, which is mediated by specific GEFs and GAPs. For example, upon EGF stimulation, BPGAP1/ARHGAP8 recruits the Rac GEF VAV1, which activates Rac1, required for cell polarisation and spreading, while simultaneously downregulating RhoA activity. Thus, this prometastatic RhoGAP, BPGAP1, acts as a dual-function scaffold that synchronizes Rac1 signalling with RhoA inactivation necessary for cell motility via lamellipodia formation (Wong et al., 2023). Interestingly, a study shows that RhoA signalling inhibits lamellipodia formation through two possible mechanisms in high-pressure cells. By acting upstream of Rac1, RhoA-mediated actomyosin contractility increases the intracellular pressure, thereby inhibiting lamellipodia formation. Secondly, by acting downstream to Rac1, RhoA disrupts lamellipodium via muscle myosin II (NMII) (Patel et al., 2021). Additionally, RhoC, another member of the Rho subfamily, decreases actin polymerization and increases actomyosin contractility via ROCK-activated LIMK, which pulls the lamellipodium rearwards. At the leading edge, upon EGF stimulation, RhoC causes acute lamellipodium extension by inhibiting cofilin activity, which severs the actin polymerization driven by the Arp2/3 complex (Patel et al., 2021). Thus, reduced levels of RhoA, RhoC or ROCK have been observed to be associated with numerous large lamellipodia formation and defective migration. This shows that Rho/Rock signalling and actomyosin contractility play a significant role in turning off lamellipodia in regions other than the leading edge, and in maintaining a balance between actomyosin contractility and actin polymerization in lamellipodia formation. MiR-490-3p, a microRNA involved in decreasing RhoA/ROCK signalling, was found to be significantly low in lung adenocarcinoma cells and tissues, which favoured migration and tumour growth (Zhao et al., 2023).

Apart from lamellipodia and filopodia Rho GTPase signalling aids in formation of various other protrusive structures. Cdc42, along with Rho, can also be involved in the formation of invadopodia under the stimulation of MMP17 (also known as MT4-MMP) for protrusion-dependent amoeboid movement of head and neck cancer cells (Yan et al., 2020). Various proteins, such as FYCO1, TRPV4, and LMP1, contribute to invadopodia formation in human HeLa cells, glioblastoma cells, and nasopharyngeal carcinoma cells by activating the Cdc42/N-WASP pathway (Sun et al., 2022; Yang et al., 2020; Tang et al., 2023). Translational activation of Cdc42 and RhoA by eIF3a leads to the formation of pseudopodial protrusions and actin remodelling, helping colorectal cancer cell migration (Mei et al., 2022). In 3D migration studies, Rho/ROCK signalling drives the lobopodian formation. The type of protrusive structure formed is dependent on matrix factors; pliable matrices favour Rho/ROCK-mediated lobopodian formation (Ridley, 2015). Another protrusive structure known as “microtentacles” has been identified, and it has been proposed that they can promote cell adhesion, motility and invasion (Wolf et al., 2020). Cycling of active and inactive forms of Cdc42 regulates septins, a family of guanine-nucleotide binding proteins associated with the actin cytoskeleton, which in turn regulate the formation of microtentacles that control cell metastasis (Østevold et al., 2017).

For a comprehensive understanding of Rho GTPase roles in protrusive structure formation, readers are referred to Warner *et al.*, who have delved deeper into the underlying signalling mechanisms. (Warner et al., 2019).

### 2.3 Role of Rho GTPases in formation of adhesive structures related to cell migration

Adhesive structures, such as FAs, are formed and disassembled in a coordinated fashion, allowing the cell to maintain its attachment to the ECM while allowing movement. Assembly of FAs involves binding integrin receptors on the cell surface to ECM proteins, recruitment of cytoplasmic proteins such as talin and vinculin stabilizing adhesion complex, and the recruitment and activation of FAK to the adhesion complex triggering downstream signalling pathways that regulate cytoskeletal dynamics and adhesion turnover (Mishra and Manavathi, 2021; Mavrakis and Juanes, 2023). All Rho GTPases Rho, Rac and Cdc42, are involved in the formation and assembly of focal complexes, which are associated with the membrane protrusions at the leading edge of the cell (Nobes and Hall, 1995). Cdc42 signalling activates Rac1, which then activates downstream effectors to facilitate migration and the formation of new adhesions. In turn, Rac1 signalling can activate RhoA, which then promotes the formation of stress fibres and focal adhesions, both of which are essential for cell contraction and adhesion (Nobes and Hall, 1995). The sequential activation of Rac1 and RhoA downstream of Cdc42 is crucial for coordinating the various steps of cell migration and adhesion.

Integrin α2β1-mediated signalling pathways involve the activation of several intracellular signalling proteins, including Rac, Cdc42, and PAK. Studies have shown that integrin α2β1 engagement promotes Rac1 activation, which in turn leads to the activation of PAK and the subsequent induction of platelet spreading (Suzuki-Inoue et al., 2001). It is further shown that FAK facilitates activation of Rac1 via βPIX, targeting activated Rac1 to FA and regulates actin dynamics and FA turnover (Chang et al., 2007). Rac1 activation has been shown to promote microtubule (MT) plus end growth and turnover, however, inhibition of PAK has been shown to block Rac1(Q61L)-induced MT growth in PtK1 cells that highlight the importance of the Rac1-PAK signalling pathway (Wittmann et al., 2003). Additionally, MCAK (mitotic centromere-associated kinesin) is involved in the transport of Rac1 and other factors along MTs to the leading edge of the cell. MCAK is located at the plus tips of MTs, promoting the dynamic turnover of these MT ends and generating a dynamic array of MTs that can interact with FAs and stimulate their turnover. This turnover, in turn, allows the cell body to translocate forward. It also enhances proper centrosome positioning toward the leading edge of the cell (Zong et al., 2021). Recently, it was demonstrated that Rac1 induces the assembly of septin filaments close to FAs, which promote non-centrosomal MT growth into FAs and inhibit MCAK-associated MT disassembly, maintaining intact MT plus ends proximal to FAs (Merenich et al., 2022). A motor protein, MII HC, heavy chain of myosin II, also generates a contractile force in cells assisting migration. Rac1-mediated PKC-dependent phosphorylation of serine 1916 on the MIIA heavy chain (HC) increases integrin-dependent capture and assembly of MIIA (myosin IIA) minifilaments in mature FAs and facilitates FA assembly (Pasapera et al., 2015). Another motor protein, Myo1e, regulates the expression and localization of integrins and adhesion molecules in membrane protrusions. It enhances the phosphorylation of FAK and control the migration of B lymphocytes through the PI3K–AKT–Rac1 signalling pathway (Girón-Pérez et al., 2020). Recently, Peng et al., have shown that NMII isoforms play two distinct roles in establishing directional cell migration on stiff substrates. Stiff substrates activate NMIIA at the leading edge via Rac1/p-PAK1/pS1916-NMIIA signalling pathway promoting directional cell motility, actin polymerization and FA dynamics. On the other hand, NMIIB is distributed in the perinuclear region, polarizing traction force distribution via PKCζ/pS1935-NMIIB signalling pathway (Peng et al., 2022).

It has been shown that constitutive activation of Rac1 prevents the maturation of nascent adhesions to FAs. It means that Rac1 promotes assembly of nascent adhesions but block maturations to enhance migration. When Rac1 is inactivated and RhoA is activated, mature FAs are formed suggesting that a spatial regulation of Rac1 and RhoA activity is required to provide the necessary traction and stability for the cell to move (Brown et al., 2005; Vitali et al., 2019). Additionally, paxillin recruitment of the PKL-PIX-PAK complex to FAs promote the transition from Rho-to Rac-mediated assembly of focal contacts, which facilitates membrane protrusion and cell migration (Chen et al., 2005). Furthermore, Arf6-GTP facilitates activation of Rac1, which promotes the formation of dynamic IACs and prevent them from maturing too quickly. An α4 integrin–paxillin–Arf-GAP complex mediate spatial polarization of Rac1 activation to the leading edge of migrating cells driving directional migration (Nishiya et al., 2005). Localization of active Rac1 to cell protrusions promotes and stabilizes highly aligned FAs and associated F-actin, facilitating cellular protrusions in the direction of ECM alignment and aiding in directed cell migration (Wang WY. et al., 2018). It has been reported recently that DDR1b colocalizes with talin and integrins to FAs promotes Rac activation by inhibiting BCR GAP activity which in turn enhances HEK293T cells migration.

Furthermore, hepatitis B virus-associated HCC has high expression of IQGAP1, which enhances the expression of Rac1. This, in turn, leads to increased levels of ROS within the cells. The increased ROS activates the phosphorylation of Src kinase, ultimately leading to the activation of FAK signalling (Mo et al., 2020). Secreted proteins like Wnt5b are also involved in a wide range of cellular processes, including embryonic development. Wnt5b has been shown to activate Rac1 and Cdc42 through downstream effectors, such as FAK and Dsh/Daam1, and promote the convergence and extension of cells during gastrulation, a crucial step in embryonic development (Hung et al., 2020). Recently, MAGI1, a protein with membrane-associated guanylate kinase, WW, and PDZ domains, has been identified as a new component of FAs. This protein is found to co-localise with phospho-paxillin, β3-integrin, and talin 1 at mature and large FAs in endothelial cells, as well as with α-4-actinin at actin stress fibres and focal adhesions. Additionally, MAGI1 plays a role in promoting endothelial cell adhesion to extracellular matrix proteins and activating the RhoA and Rac1 signalling pathways (Alday-Parejo et al., 2021).

Tip cells are specialized endothelial cells that are found at the growing tips of blood vessels. During the endothelial tip cells invasion, there is a balance between dactylopodia and filopodia protrusions which is regulated by myosin IIA (NMIIA) and actin-related protein 2/3 (Arp2/3). Dactylopodia are filopodia type finger-like extensions of the PM associated with membrane ruffling activity. When NMIIA is ablated, excessive dactylopodia formation occurs at the expense of filopodia. Conversely, when Arp2/3 is ablated, dactylopodia development is prevented and excessive filopodia formation occurs.

Additionally, the study reveals that NMIIA inhibits Rac1-dependent activation of Arp2/3 by regulating the maturation state of FA, which is important for cell adhesion and migration (Figueiredo et al., 2021). Mechanotransduction has been shown to play a crucial role in angiogenesis and the formation of tip cells, specifically the endothelial cells involved in this process. The stiffness of the ECM induces phosphorylation in paxillin (p-PXN) in FAs. The transduced tension of the actin cytoskeleton via active Rac1 contributes to the transfer of YAP from cytoplasm to nucleus which promotes tip cell specification through mechanotransduction (Guo et al., 2022). A recent study has shown that the transcriptional coactivator YAP1 and TEADs enhance the expression of TIAM1 and promotes Rac1 activation and invadopodia formation in breast cancer. These findings suggest that the Hippo signalling pathway and YAP1 activity may be important therapeutic targets to inhibit invadopodia formation (Shen et al., 2022). Moreover, GCN2 detects amino acid levels through uncharged transfer RNAs (tRNAs). Wounding activates GCN2, which aids wound healing by increasing cystine transporters to maintain cysteine levels and triggering ROS production through NOX enzymes. This connects translational control and actin remodelling during keratinocyte migration and wound healing through Rac1 activation (Miles et al., 2021).

Assembly of FAs is associated with changes in tyrosine phosphorylation of FAK and paxillin which is a RhoA dependent process (Barry and Critchley, 1994). Myosin 1B (MYO1B), a member of the myosin superfamily that also includes other myosins, is crucial for cell migration and motility. By increasing the activation of RhoA, MYO1B was shown to facilitate F-actin rearrangement via the ROCK2/LIMK/Cofilin axis along with enhancing the formation of FAs (Xie et al., 2021). By detecting changes in the content and mechanics of the ECM, hippo effectors YAP/TAZ function as on-off mechanosensing switches. In fact, a recent study has revealed that YAP functions downstream of the RhoA pathway to regulate the FA assembly. Overexpression of RhoA caused nuclear shuttling of YAP, which directly regulated the expression of various FA-related genes (Nardone et al., 2017). In addition, biochemically, when a gain-of-function mutation was created in RhoA (RhoAY42C), in which GTP hydrolysis was impaired, it was noted to be associated with increased interaction with ROCK, resulting in enhanced actin rearrangements and focal adhesion kinase activation (Zhang H. et al., 2020).

The roles of Cdc42 are nominal as compared to other Rho GTPases in formation of adhesive structures. Cdc42 is known to interact with lysosome‐associated protein transmembrane 4B (LAPTM4B) and localise it to filopodia where it stabilises the filopodia and stimulates FA initiation and dynamics with its downstream signalling (Liu et al., 2022). A microRNA miR-497, has been shown to inhibit Cdc42 via the ITGB1/FAK/PXN/AKT axis leading to inhibition of FA formation and phosphorylation of its two key proteins FAK and Paxillin which could be rescued by Cdc42 overexpression (Zhang L. et al., 2021). Cdc42 is known to affect FAK phosphorylation, as well as the phosphorylation of GSK3β and β-catenin, thereby altering the adhesion and polarised movement of human dental pulp stem cells (Li et al., 2018).

### 2.4 Rho GTPases in matrix metalloprotease (MMP) secretion as a prerequisite for cell migration

**FIGURE 3:**
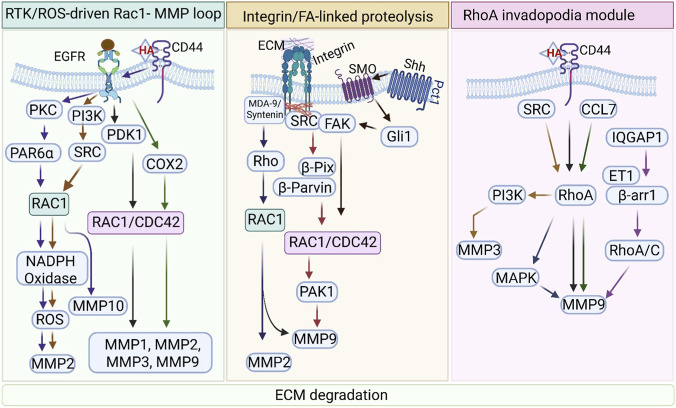
Rho GTPase–mediated matrix metalloproteinase (MMP) regulation and extracellular matrix (ECM) degradation. Extracellular matrix (ECM) degradation during cell migration is regulated by distinct but interconnected Rho GTPase–centered signalling modules, which integrate receptor signalling, cytoskeletal dynamics, and protease activation. The figure is organized into three mechanistic themes: RTK/ROS-driven Rac1–MMP loops, integrin/focal adhesion (FA)-linked proteolysis, and RhoA-dependent invadopodia modules, all converging on ECM degradation. Left: RTK/ROS-driven Rac1–MMP loop: Activation of receptor tyrosine kinases (RTKs), particularly EGFR, in conjunction with CD44–hyaluronic acid (HA) signalling, engages PI3K, PKC, SRC, and PDK1 pathways, leading to activation of Rac1 and Rac1/Cdc42 signalling axes. Rac1 stimulates NADPH oxidase–dependent ROS production, which amplifies downstream signalling and promotes transcriptional and post-translational activation of MMPs, including MMP1, MMP2, MMP3, MMP9, and MMP10. Additional mediators such as COX2 and PAR6α further reinforce Rac1-dependent invasive signalling, establishing a feed-forward loop coupling RTK activation to protease production. Middle: Integrin/FA-linked proteolysis: Cell–matrix interactions mediated by integrins and extracellular matrix (ECM) components activate FAK/SRC signalling, which integrates with Hedgehog (Shh–SMO–Gli1) signalling and adaptor proteins such as MDA-9/syntenin. These pathways converge on Rho GTPase regulators, including β-PIX and β-parvin, to activate Rac1 and Cdc42, promoting cytoskeletal remodeling via PAK1 and facilitating targeted protease activity. This module emphasizes the spatial regulation of proteolysis, including MT1-MMP–dependent matrix degradation and localized activation of MMP2 and MMP9, linking focal adhesion dynamics to ECM remodeling. Right: RhoA invadopodia module: RhoA signalling governs the formation and maturation of invadopodia, specialized actin-rich structures responsible for localized ECM degradation. Upstream signals from CD44/HA, SRC, and chemokines such as CCL7 activate RhoA, which integrates with PI3K, MAPK, and IQGAP1 scaffolding networks. The endothelin-1 (ET1)/β-arrestin1 (β-arr1) axis further promotes RhoA/C activation, facilitating invadopodia maturation and targeted delivery of proteases. These pathways drive the expression and localization of key proteases, including MMP3 and MMP9, enabling efficient matrix degradation.

Remodeling of the extracellular matrix (ECM) is often a prerequisite for efficient cell migration, particularly in invasive or confined environments. A major component of this process is the expression, activation, and localized deployment of matrix metalloproteinases (MMPs), which cleave ECM components and thereby facilitate cell movement (Winkler et al., 2020). Rho family GTPases are central to this process, not only because they control actin remodeling and cell polarity, but also because they link receptor signalling to protease production, trafficking, and the formation of invasive structures (Figure 3). The studies summarized below indicate that the contribution of Rho GTPases to matrix degradation can be organized into three broad mechanistic modules: RTK/ROS-driven Rac1–MMP loops, integrin/focal adhesion-linked proteolysis, and RhoA-dependent invadopodia modules.

#### 2.4.1 RTK/ROS-driven Rac1–MMP loop

One major mechanism by which Rho GTPases promote matrix degradation is through Rac1-dependent signalling downstream of receptor tyrosine kinases, particularly EGFR. EGFR activation promotes invasion in multiple tumour types by coupling PI3K and Src signalling to Rac1 activation, which in turn stimulates NADPH oxidase-derived ROS and enhances MMP-2 secretion and activation, as shown in pancreatic cancer (Binker et al., 2009). Similarly, EGF-induced migration and invasion in breast cancer can be amplified through Rac1-dependent MMP-9 activation, as demonstrated for psoriasin/S100A7 in ERα-negative breast cancer (Al-Haddad et al., 1999). Additional studies in head and neck squamous cell carcinoma further show that EGF signalling can induce COX-2-dependent expression of MMP-1, MMP-2, MMP-3, and MMP-9, together with fibronectin, in association with Rac1/Cdc42 activation (Hsu et al., 2015). In the same context, PDK1 promotes metastasis by enhancing the expression of these MMPs (MMP-1, MMP-2, MMP-3, MMP-9) via EGF/FN/Rac1/Cdc42 signalling (Hsu et al., 2017). These examples support a recurrent theme in which EGFR-centered signalling converges on Rac1 to couple migratory signalling with protease induction.

This RTK–Rac1–MMP axis is further reinforced by receptor crosstalk and redox amplification. In melanoma cells, hyaluronic acid-dependent CD44–EGFR interaction activates PKC signalling involving Rac1, leading to production of ROS, FAK, and MMP-2 and enhanced motility (Kim et al., 2008). Conversely, pharmacologic inhibition of Rac1 (Rhein) in ovarian cancer reduces expression of MMP2, MMP3, MMP9, and MMP19, together with suppression of the Rac1/ROS/MAPK/AP-1 pathway, supporting a causal role for Rac1 in MMP regulation (Zhou et al., 2017). The same general logic is seen in non-small cell lung cancer, where PKCι–Par6α-mediated Rac1 activation drives invasion through upregulation of MMP-10 (Frederick et al., 2008). Likewise, in hepatocellular carcinoma, downregulation of Annexin A5 suppresses Rac1-and MEK–ERK-associated signalling together with MMP-9, whereas in colorectal cancer Plastin-1–IQGAP1/Rac1/ERK signalling enhances MMP2 and MMP9 expression (Sun et al., 2018; Zhang T. et al., 2020). These studies collectively argue that Rac1 functions as a central signalling hub coupling RTK-associated pathways to transcriptional and redox-dependent MMP programs.

Rac1-dependent control of matrix degradation also extends beyond classical EGFR signalling. CXCR4 activation enhances Rac1-dependent MMP-2 activity in bronchial and alveolar epithelial cells (Ghosh et al., 2012), while Interleukin-37 suppresses endometrial cancer migration and invasion through inhibition of the Rac1/NF-κB/MMP2 axis (Wang et al., 2021). In hepatocellular carcinoma, a reciprocal interplay between TGF-β1 and MMP8 promotes EMT through PI3K/AKT/Rac1 signalling, further illustrating how Rac1-dependent pathways integrate receptor signalling with protease regulation (Qin et al., 2016). Together, these findings support the idea that Rac1-centered signalling networks are major drivers of MMP expression and activation, especially when linked to ROS-generating pathways and RTK cross-talk.

#### 2.4.2 Integrin/focal adhesion-linked proteolysis

A second major theme is that Rho GTPases regulate matrix degradation through integrin- and focal adhesion-associated proteolytic mechanisms. In this module, the emphasis is not simply on MMP expression, but on the coupling of actin remodeling, focal adhesion dynamics, and localized protease deployment. Hedgehog signalling provides one such example: Shh/Gli1 upregulates integrin β1 and induces F-actin formation through FAK/Src and Rac1/Cdc42 signalling, while also promoting MMP-associated adherens junction disruption (Oh et al., 2018). In triple-negative breast cancer, the focal adhesion protein actopaxin (α-parvin) forms a complex with β-PIX and PAK1—a Rac1/Cdc42 GEF-effector pair—to regulate matrix degradation, linking focal adhesion scaffolds directly to proteolytic function (Pignatelli et al., 2012). Similarly, Rab8 promotes Rac1-and TIAM1-dependent cortical actin polymerization and coordinates microtubule-dependent focal adhesion disassembly through MT1-MMP (MMP-14) and calpain, providing a direct link between Rac1-driven membrane dynamics and membrane-tethered protease activity (Bravo-Cordero et al., 2016). Additional studies reinforce the importance of integrin-associated signalling in positioning proteolysis within the migrating cell. MDA-9/syntenin influences neuroblastoma migration through an integrin–Src–Rho–Rac axis (Bhoopathi et al., 2019), while CRISPR-mediated depletion of a metalloprotease ADAMTS-1 results in Cdc42 polarization at the leading edge and increased migration and invasion, implicating localized protease control in polarity-associated invasive behaviour (de Assis Lima et al., 2021). In preeclampsia-associated trophoblast invasion, Rac1–β-catenin signalling promotes Snail and MMP9 expression, further illustrating how focal adhesion- and actin-associated signalling can feed into MMP-dependent invasion (Fan et al., 2016). Taken together, these examples support a model in which integrin/focal adhesion-linked signalling acts as a spatial organizer of proteolysis, coordinating Rac1/Cdc42 activity, actin remodeling, and membrane protease deployment to facilitate directional matrix degradation.

#### 2.4.3 RhoA invadopodia modules

A third, conceptually distinct module centres on RhoA-dependent invadopodia formation and maturation. Invadosomes, including invadopodia and podosomes, are actin-rich protrusive structures specialized for ECM degradation, and accumulating evidence indicates that RhoA signalling is critical for their mechanical and proteolytic competence (Augoff et al., 2020). During endothelial cell migration, MMP-9 clusters colocalize with RhoA and CD44 in lamellipodia, providing early evidence that RhoA is associated with localized protease deployment (Abécassis et al., 2003). In hepatocellular carcinoma, autocrine motility factor/phosphoglucose isomerase induces migration through Src–RhoA–PI3K-dependent transactivation of MMP-3, whereas inhibition of RhoA/MAPK signalling reduces MMP-9 secretion and suppresses invasion in nasopharyngeal carcinoma (Shih et al., 2008). These examples support a role for RhoA in regulating the expression and secretion of specific metalloproteinases linked to invasive behaviour.

The role of RhoA becomes especially clear in the context of invadopodia maturation. In lung and colon cancer cells, CCL7 signals through RhoA to target MMP-9 to the invadopodia complex and promote invadopodia maturation (Qi et al., 2020). Likewise, IQGAP1, together with the endothelin-1/β-arrestin1 network, is required for invadopodia function, and in this setting, Rac1 is suppressed while RhoA/C signalling is promoted, thereby supporting correct invasive structure formation and ECM degradation through MMP secretion (Chellini et al., 2019). Mechanical signalling converges on this same module: matrix stiffness can be transduced through integrin β/RhoA/ROCK1 and Piezo1/Ca2+/MLCK signalling to drive MLC2-dependent actomyosin contractility, which is required for invadopodia formation and matrix-reinforced migration (Zhang et al., 2024). Similarly, LPA-induced prostate cancer progression is driven by concurrent activation of RhoA and NF-κB, enhancing invadopodia formation and proteolytic capacity (Hwang et al., 2016).

Further evidence suggests that invadopodia progression is governed by a specialized PAK hierarchy in which PAK1 initiates structural assembly, whereas PAK4 promotes functional maturation by recruiting PDZ-RhoGEF to activate RhoA-mediated contractility required for invasive persistence (Nicholas et al., 2019). In addition, RhoA–VAV1 interaction enhances invasion specifically through MMP-9, while growth/differentiation factor-5 promotes epidermal stem cell migration through a RhoA–MMP signalling axis (Nakamura et al., 2023). Other studies extend this logic to matrix-associated proteins and metalloproteases, including type VIII collagen/β1 integrin signalling and ADAM8-associated invasion, both of which correlate with activation of RhoA, FAK, MAPK, Src, and β1 integrin (Fagan-Solis et al., 2013; Awan et al., 2021). Collectively, these observations position RhoA not merely as a regulator of contractility, but as a central organizer of the mechanical, structural, and proteolytic events required for invadopodia-dependent invasion.

Taken together, matrix degradation during migration is coordinated by three interdependent Rho GTPase-centered modules: Rac1-dominant RTK/ROS pathways drive MMP expression and activation; integrin/focal adhesion-linked pathways spatially organize proteolysis at sites of matrix engagement; and RhoA-dependent invadopodia programs provide the contractile and structural framework necessary for persistent ECM degradation.

### 2.5 Role of Rho GTPases in cell body contraction (actomyosin contractility) and tail detachment in cell migration

Cell body contraction during migration is mediated by actomyosin contractility. RhoA through its concurrent activation of the ROCK kinase and of the formin mDIA is assumed to be the driver of actomyosin contractility and subsequently rear retraction in cell migration (Amano et al., 2000). The non-muscle Myosin light chain is phosphorylated and activated by the kinase ROCK, and actin bundles formed by mDIA’s actin polymerization serve as a substrate for Myosin II to bind to. Myosin II’s affinity for actin filaments and its particular location at the rear of migrating cells at the time of migration cause the contractility that pushes the cells forward. Several MS ion channels have been connected to the regulation of RhoA activity and, consequently, of myosin II. In kidney podocytes and fibroblasts, TRPC6 joins forces with RhoA to create a molecular complex. By the calcium-dependent Pyk2 and an increase in RhoA activity, TRPC6-mediated Ca^2+^ influx prevents cell migration by activating myosin II across the cell (Canales Coutiño and Mayor, 2021). Activin B, a member of TGFβ superfamily was shown to induce a new signalling cascade RhoA/ROCK/LIMK2/Cofilin that controls the development of actin stress fibres for the migration of bone marrow derived mesenchymal stem cells (Wang X. et al., 2017). Interestingly, Ezrin a cytoskeletal crosslinker is proven to positively regulate RhoA but not Rac1 to promote formation of actin stress fibres in ovarian cancer cells (Li MJ. et al., 2021). For the cell to migrate the focal complexes in the rear must dissociate. When there is reduced growth factor signalling, protein kinase N a effector of RhoA and target of mTORC2 senses reduced growth factor and then synergizes with an FA disassembly factor called as DEPDC1B resulting in the induction of Phosphatidylinositol-3,4-bisphosphate, a membrane lipid that promotes FA disassembly and promotes the turnover of stress fibres by recruiting a Rho-GAP, ARAP3 (Posor et al., 2022). Upon vimentin depletion, the microtubule-associated GEF-H1 is phosphorylated on Ser886 which enhances RhoA activity and actin stress fibre assembly. This suggests that intermediate filaments control contractile actomyosin bundles and may shed light on the relationship between elevated levels of vimentin expression and cancer cell motility and invasion (Jiu et al., 2017). Asymmetry in FA dynamics along the front-to-rear is essential for trailing edge detachment and persistent migration. Kindlin-2, a protein involved in integrin mediated ECM-cell adhesion is suggested to play role in FA disassembly in the rear and promote actomyosin contractility at the FAs leading to tail detachment (Liu J. et al., 2021). Also, Prickle 1 a protein involved in planar cell polarity maintenance preferentially accumulates at the retraction site of migrating cell by localizing in close proximity to paxillin at FA and regulating their disassembly (Lim et al., 2016). Calcium release-activated calcium modulator 2 (ORAI2), a subunit of the calcium channel, was shown to favour gastric cancer cell migration by involving in FA disassembly in the rear edge via FAK-mediated MAPK/ERK activation (Wu et al., 2021). In addition, knockdown of the Tau protein in the glioblastoma cell line was observed to be associated with inefficient tail retraction. A decrease in tau was observed to be correlated with high levels of the active, phosphorylated form of p190RhoGAP, which inhibits Rho/ROCK signalling and FAK, leading to defective rear retraction (Breuzard et al., 2019). Interestingly, Cas (Crk-associated substrate/p130Cas/BCAR1) regulates the trailing edge retraction by decreasing the stability of FA in the rear while maintaining leading edge stability. This has been explained to localized action of phosphorylated Cas. FAs in the front contain phospho-Cas which then recruits SOCS6 a ubiquitin ligase and inhibit Cas dependent FA turnover more in the front than in rear (Teckchandani and Cooper, 2016).

## 3 Roles of Rho GTPases in collective cell migration (CCM)

Collective cell migration (CCM) refers to the coordinated movement of cells in large groups, contributing to various cellular functions such as morphogenesis during embryonic development, wound healing, angiogenesis, and cancer cell migration. CCM differs from single cell migration in that it exhibits a specific speed and direction, making it more efficient. Some CCM behaviours include having cell-cell contacts throughout migration, in which each cell coordinates the movement of its adjacent cells as well, resulting in the cells migrating as a cohesive unit. In contrast, a different type of CCM involves cells having cell-cell contacts only briefly, but the cells are loosely associated with each other. In the latter type of CCM, the individual cells of the group of loosely interacting cells have similar characteristics to those of cells in single cell migration, i.e., a front and rear polarity axis aided by molecular pathways regulated by the Rho GTPases, maintaining the polarity, which we discussed above (Mayor and Etienne-Manneville, 2016). However, in cohesive type CCM, the cell-cell contacts modify the cell behaviours. The cells at the front end of migration act as leader cells, helping to sense the external environment and dictate the nature of the migration. The cells preceding the leader cells, known as follower cells, rely on cell-cell communication from the leader cells to collectively polarise; therefore, the mechanisms in the follower cells undergoing migration might vary from the usual. Just as the signalling pathways at the rear of single cells migrating control the front of the cells, the signalling pathways in follower cells can also influence the leader cells and thereby, the CCM (Mayor and Etienne-Manneville, 2016). The complete pathways by which cells undergo CCM under the influence of Rho GTPases have been described by Mayor and Etienne-Manneville, as well as Zegers and Friedl, in two extensive reviews (Zegers and Friedl, 2014; Mayor and Etienne-Manneville, 2016). We will discuss the advances in these processes related to CCM controlled by RhoGTPases since then, briefly.

The leaders in CCM interact with the extracellular environment more or less similarly as is expected from a single cell undergoing migration-i.e., the integrins engage with the ECM leading to the recruitment/activation of Cdc42 and Rac via GEFs associated with the respective adaptor proteins and kinases. A research group studied the effects on CCM upon P-cadherin expression by performing an *in-vitro* directional migration assay on myoblasts. It was shown that P-cadherin recruited the GEF β-PIX, that led to Cdc42 activation, followed by changes in cell polarity, membrane protrusions and FAs to collective movement of the entire monolayer. Plithotaxis is a process in coordinated migration in which the cell orients its movement in the direction where the normal stress is highest and the shear stress is the least (Trepat and Fredberg, 2011). The said study found that the P-cadherin/β-PIX/Cdc42 pathway promotes CCM through plithotaxis (Plutoni et al., 2016). In 2020, a group proposed integrin/integrin antagonism to be a universal mechanism in controlling different facets of CCM. They showed that a reciprocal β1/β3 antagonism in different collective cell processes controlled RhoA activity via kindlin-2 projecting its effects on cell-cell adhesion, cell spreading, contractility and regulation of plasticity like in EMT (van der Bijl et al., 2020).

Next, the leader cells form membrane protrusions. A study elucidates the working mechanism between co-attraction and contact inhibition via Rho GTPase for spontaneous CCM in neural crest cells. They showed that co-attraction and contact inhibition together led to persistent polarity in a neural crest cells cluster by suppressing random activation of Rac1 thereby suppressing protrusion formation (Merchant et al., 2018). A RhoA GEF, ARHGEF40, has been shown to localise to cell-cell contact regions in cells undergoing CCM and leads to accumulation of keratin-8/keratin-18 (K8/K18) filaments in the front edge of the cells and also acts as a brake for CCM by creating pull-back forces at cell-cell junctions via RhoA-ROCK axis (Isozaki et al., 2020). In *Drosophila* border cell CCM, it was found that Cdc42 is localised to the border cells under the action of another small GTPase Rab11 and regulates cell-cell communication as well as formation of protrusions (Colombié et al., 2017). Supracellular actin cables and border cell protrusions are essential for direction and coordination in CCM and have been shown to have two switchable pools of Rac1 that integrate both the processes (Zhou S. et al., 2022). A protein, HAX1, is involved in regulating cell-cell junctions, epithelial cell layer integrity, and cell-substrate adhesion via RhoA and septin signalling, which modulates actomyosin contractility (Balcerak et al., 2019). A Scrib/Cdep/Rac pathway has been recognised for cell crawling and coordinated CCM by studies done on *Drosophila* border cells (Campanale et al., 2022). A study showed that during collective cell migration, contractile forces drove the microtubule-dependent peripheral accumulation of lysosomes in emerging leader cells. These repositioned lysosomes functioned as an intracellular platform by associating with Rac1 at the leading edge, thereby regulating local Rac1 activity to trigger actin polymerization and lamellipodium formation (Marwaha et al., 2025).

A modelling study found that, unlike single-cell migration, in CCM, Rho GTPase activity controls the mechanical contraction and extension of cells in a wave that synchronises throughout the tissue. The intensity of the force also varies at the leading and trailing ends, with higher junctional forces associated with faster migration (Bui et al., 2019). The zebrafish posterior lateral line primordium (pLLP) serves as a premier *in vivo* model for understanding how discrete intracellular signals are integrated to produce cohesive tissue-level movement. Recent live-imaging studies have revealed that collective motility in the pLLP is fundamentally dependent on pulsatile RhoA signalling and subsequent Myosin II activation, specifically localized at the basal surface of the primordium (Qian et al., 2024).

This study demonstrated that during collective cell migration in MDCK cells, front-to-rear propagation of EGFR-Ras-ERK signalling occurred independently of EGFR ligand gradients, with Rac1 and Rab5A establishing front–rear polarity. EGFR and Ras provided temporal cues via pulsatile waves from leader cells, while Rab5A facilitated Rac1 activation at the front, positioning Ras and Rab5 as integrators of spatial and temporal inputs (Jikko et al., 2025). The dynamics between the follower and leader cells in CCM goes beyond just RhoGTPase signalling and a broader aspect of the same has been provided by Qin et al., in 2021 followed by Wan et al., very recently in 2025 (Qin et al., 2021; Wan et al., 2025).

## 4 Other Rho GTPases and their potential contributions to cell migration

While Rac1, RhoA and Cdc42 remain the most well characterised amongst all Rho GTPases with respect to cell migration, other Rho GTPases also have roles in the same and some, significantly so.

### 4.1 RhoB

The different roles of RhoA, RhoB and RhoC in cancer cell migration have been discussed by Ridley (Ridley, 2013). RhoA and RhoB have been shown to have an inverse relationship. In two separate studies done in 2018 and 2020, an increased RhoA led to decreased RhoB protein expression which was shown to regulate the migratory properties and invasiveness in skin and breast cancer (García-Mariscal et al., 2018; Privat et al., 2020). A RhoB/ROCK/PTEN axis was elucidated in breast and brain cancer cells that led to increase in amoeboid migration, durotaxis and topotaxis (Wieland et al., 2021). RhoB acts as a tumour suppressor in many cancers and quite an amount of research has been done on the role of micro RNAs like miR-19a, miR-223, miR-663a and miR21 controlling RhoB and its consequent functions on cell migration (Li et al., 2019; Hu et al., 2022a; Yao X. et al., 2020; Bai et al., 2023). Another small GTPase, Arf6, has been shown to recruit RhoB to the PM and mitochondrial membrane, and the depletion of Arf6 leads to endolysosomal degradation of RhoB, leading to defective FAs (Zaoui et al., 2019). It has also been shown that PM -localized RhoB is endocytosed by Rab5-positive vesicles, recycled back to the same location via Rab11-positive endosomal vesicles under the regulation of KIF13A, where it initiates the formation of membrane blebs via ROCK and Myosin II, leading to amoeboid cell migration (Gong et al., 2018).

### 4.2 Rho C

RhoC is known to participate extensively in both 2D and 3D cell migration and has a role in practically all steps of 2D cell migration, starting from cell polarisation, to adhesion, cell body contraction and tail retraction, all of which have been extensively described by Lou et al. (2021). Since then, some research has been done on the role of RhoC in cell migration in different cancers. RhoC has roles in oral squamous cell carcinoma (OSCC) cell invasion via regulation of HMGA2 expression and also mediating RhoC/Src pathway (Gao et al., 2021; Yin et al., 2021). In another study on glioblastoma, RhoC/Cofilin pathway has been shown to increase cell migration and is downregulated by LACTB (Hu et al., 2022b). RhoC is also involved directly in cell migration and invasion capabilities of colon cancer cells via the PTEN/FOXO1 axis (Yang et al., 2023). A long non-coding RNA lnc00892 has been shown to reduce transcription of nucleolin, the absence of which then leads to destabilisation of RhoA/RhoC mRNA leading to a reduction in cell migration and metastasis in bladder cancer (Ren et al., 2021). Another lncRNA ZFAS1, acts as a sponger for miR-520b and miR520e leading to RhoC mediated increase in cell migration and invasion (Liu X. et al., 2023).

### 4.3 Rac2

Myotubes in *Drosophila* testis require filopodia instead of lamellipodia for collective cell migration for which they require Formins, and Rho GTPases like Rac2, Cdc42 and RhoA, apart from this Rac2 and Cdc42 were also required for cell matrix adhesion (Bischoff et al., 2021). Rac2 is also involved in the regulation of T lymphoid progenitor cells to thymus during embryogenesis in Zebra fish (Lu et al., 1950). Rac2 is also known to be upregulated in clear renal cell carcinoma and has been shown to be a potential therapeutic target in the same because of its roles in cell migration and invasion (Liu Y. et al., 2019).

### 4.4 Rac3

Rac3 has been shown to be upregulated in bladder cancer with a poor prognosis for patients. It is involved in bladder cancer cell migration and invasion by activating JAK/STAT pathway via PYCR1 (Cheng et al., 2020). Similarly, another study showed that inhibition of Rac3 lead to a decrease in migration of bladder cancer cells due to autophagy by the PI3K/AKT/mTOR pathway (Wang L. et al., 2022). Rac3 has also been found to be a transcriptional co-activator of ERα and aids in E2 mediated cell migration in ERα positive breast cancer cells (Walker et al., 2011). Rac3 has also been shown to promote cell migration of NSCLC cells by modulating AKT/NF-κB pathway in cancer associated fibroblasts which was promoted by increase in m6A level of Rac3 by METTL3 (Chen et al., 2023). A similar study showed the role of increased m6A level of Rac3 by METTL3 in rheumatoid arthritis fibroblast-like synovial cells led to increased cell motility amongst other phenotypes (Ren et al., 2025). Variants of Rac3 like p.N92K and p.F28S help in brain development associated neuronal migration as well (Sugawara et al., 2025; Nishikawa et al., 2023).

### 4.5 RhoG

RhoG was found to be an inducer of lamellipodia formation (Aspenström et al., 2004). It is also a known upstream regulator of Rac via ELMO/Dock4 or Dock180 complex (Katoh et al., 2006; Katoh and Negishi, 2003; Hiramoto et al., 2006). A member of the Eph receptor family, EphA2 is known to promote ligand independent promotion of cancer cell invasion and motility. The mechanism behind this was elucidated in which EGF stimulation of EGFR led to activation of EphA2 which along with Ephexin4 activates RhoG. RhoG forms a complex of ELMO2 and Dock4 (a Rac GEF) along with EphA2 at cortactin rich protrusions leading to Rac activation and thereby cell migration (Hiramoto-Yamaki et al., 2010). It was also shown that though RhoG is not essential for integrin mediated cell spreading, it did have roles related to Rac mediated cell migration and also a constitutively active RhoG led to formation of membrane ruffles in both Rac dependent and independent manner (Meller et al., 2008). In a study done on salivary adenoid cystic carcinoma (SACC), they identified that RhoG/Rac contributed in the promotion of SACC cell migration at least partially via epiregulin/Src/AKT/ERK1/2 pathway (Xu et al., 2020). Since RhoG/Rac signalling plays a crucial role in cell migration, studies have been conducted to identify small-molecule inhibitors. These studies have also explored the roles of RhoG in cancer progression (Dipankar et al., 2023; Dasari et al., 2017). There is a bit of research done on the GEFs related to RhoG. SGEF, a member of the Ephexin subfamily of RhoGEFs, activates RhoG to promote cell migration; however, Src suppresses this activity through tyrosine phosphorylation. (Okuyama et al., 2016). Actin rich structures called circular dorsal ruffles are formed on the dorsal surface of various mammalian cells upon growth factor stimulation and can have functions in cell migration. It was found that RhoG and its GEF, Trio, play roles in CDR dynamics via size alteration, leading to effects on cell migration and micropinocytosis (Valdivia et al., 2017).

### 4.6 RhoJ

RhoJ has roles in migration of endothelial progenitor cells in early embryogenesis that are involved in the formation of the primary vascular plexus via ETV2 (Ets variant transcription factor 2) that binds to RhoJ’s promoter region (Singh et al., 2020). Activated RhoJ interacts with GIT1/2 that in turn interacts with β-PIX and paxillin at the FAs, leading to FA disassembly and increased migration via Rac and Cdc42 activation (Wilson et al., 2014). VEGF activation of RhoJ leads to its FA localisation, endothelial cell migration and roles in actomyosin contractility and FA numbers (Kaur et al., 2011). RhoJ has been shown to have considerable roles in cancer cell migration by various pathways. RhoJ also has roles in aiding the metastatic potential of breast cancer cells and is upregulated in invasive breast cancer cells compared to benign ones. Elevated RhoJ expression promotes invasion and metastasis in EMT-subtype gastric cancer via activation of the IL-6/STAT3 signalling pathway, indicating that RhoJ represents a potential biomarker and therapeutic target for this tumour subtype. An ERG1-MKL1 axis promoted by TGFβ was shown to lead to the transactivation of RhoJ, resulting in metastasis, and was considered a potential therapeutic target (Chen B. et al., 2020). In angiogenesis-associated cell migration, RhoJ is contextually influenced by VEGF and Sema3E, respectively, through different pathways, affecting forward and reverse endothelial cell migration (Fukushima et al., 2020). RhoJ has also been associated with an increase in invasiveness and metastasis in gastric cancer, glioblastoma and melanoma cells, mostly affecting the cytoskeleton dynamics (Kim et al., 2016; Wang M. et al., 2020; Ho et al., 2013). Recent studies have also shown a decrease in migration head and neck squamous cell carcinoma (HNSCC) drug-tolerant persister cells upon RhoJ knockdown (Ling et al., 2026).

### 4.7 Rnd1

Rnd1 has been known to be involved in actin cytoskeleton rearrangement and migration and invasion of cells (Nobes et al., 1998). It has been shown to both promote and inhibit cell migration by different studies. Rnd1 is found to be upregulated in esophageal squamous cell carcinoma and can aid in cell migration via ERK signalling (Xiang et al., 2016). Even in breast cancer it has been seen that overexpression of Oct4 leads to downregulation of Rnd1 and a loss in metastatic potential of breast cancer cells (Shen et al., 2014). However, various other studies have shown Rnd1 to be involved in suppression of metastasis. A study on lung squamous cell carcinoma cells showed Rnd1 to be a tumour suppressor and its presence hampered cell migration (Zhou Y. et al., 2022). In angiogenesis, Notch signalling plays a major role in regulating cell migration and it was shown that Notch transcriptionally regulated Rnd1 expression for suppression of endothelial cell migration (Swaminathan et al., 2022). In hepatocellular carcinoma (HCC) Rnd1 is involved in suppression of metastasis and invasion by negatively regulating EMT via deactivation of RhoA/Raf/MEK/ERK signalling pathway (Qin et al., 2018). Therefore, reduced expression of Rnd1 in HCC is seen more aggressive forms of the disease and promoted metastasis and invasion via acting on Rac pathway as well (Komatsu et al., 2017). Similarly in glioblastoma Rnd1 was shown to inhibit EMT and migration via modulating AKT/GSK3-beta pathway (Sun et al., 2024).

### 4.8 Rnd2

Rnd2 seems to have most roles in radial migration in brain development. During neurogenesis in the embryonic cerebral cortex, the proneural protein neurogenin 2 (Neurog2) plays a direct role in inducing Rnd2 helping in radial migration of neurons (Heng et al., 2008). Another protein Bacurd2 was shown to interact with Rnd2 and aid in radial migration of neurons (Gladwyn-Ng et al., 2015). Repressed Rnd2 expression by COUP-TFI also promoted radial migration in callosal projection neurons during corticogenesis (Alfano et al., 2011). Additionally, the zinc finger transcription factor RP58 represses Rnd2, aiding in neuronal migration during cortical development (Heng et al., 1991).

### 4.9 Rnd3/RhoE

miRNAs in different disease conditions regulate Rnd3 levels to promote cell migration and invasion. For instance, miR200b inhibits EMT in HeLa cells by decreasing expression of Rnd3 (Cheng et al., 2016). Additionally, a study on preeclampsia has shown that another miRNA, miR-182-5p, reduces the levels of Rnd3, leading to a decrease in cell invasiveness and thereby aiding in disease progression (Fang et al., 2018). Epstein-Barr virus-encoded miRNA, BART2-5p, promotes metastasis of nasopharyngeal carcinoma by suppressing RhoE (Jiang et al., 2020). As mentioned earlier, Rnd2 is involved in neuronal cell migration during neurogenesis, similarly Rnd3 plays a role in neuronal migration in the brain cortex. The proneuronal factor, Ascl1 instead of Neurogog2 controls Rnd3 expression and leads to neuronal cell migration in the cortex via inhibition of RhoA (Pacary et al., 2011). The Plexin B2 receptor has been shown to interact with Rnd3, increasing RhoA activity and, consequently, enhancing cortical cell migration (Azzarelli et al., 2014). A group found that the hypoxia response protein HIF1α directly regulates the levels of RhoE in gastric cancer leading to increase in invasiveness of the cancer during hypoxia via EMT (Zhou et al., 2011). The group later went on to elucidate that the role of RhoE in increased invasiveness of gastric cancer cells came from an upregulation of CXCR4 the exact mechanisms of which warrant further investigations (Feng et al., 2013). When cells of a subtype of Rhabdomyosarcoma called alveolar rhabdomyosarcoma were grown on type I collagen, they used an amoeboid mode of invasion via RhoE/ROCK/ARHGAP25 signalling (Thuault et al., 2016). RhoE reduces cell migration by antagonising Rho/ROCK/myosin phosphatase pathway and is found to be downregulated in HCC (Ma et al., 2013). Rnd3 regulation of the cytoskeleton influences invasive 3D growth in melanoma cells (Klein and Aplin, 2009). However, an overexpression of FOXD3 in melanoma leads to decrease in cell migration due to inhibition of Rnd3 (Katiyar and Aplin, 2011). When mutant BRAF was inhibited in melanoma cells, a switch in Rnd3-RhoA signalling was involved in cell invasive properties (Klein and Higgins, 2011). A similar observation was made in mesenchymal tumour cells as well where a decreased Rnd3 expression was associated with decreased invasiveness of the cells (Belgiovine et al., 2010). In glioblastoma cells, the degradation of Snail1 protein, promoted by Rnd3, leads to the inhibition of cell migration and invasion (Liu et al., 2016). Nanog mediated downregulation of RhoE also leads to decrease in cell migration (Zhou et al., 2013). A feedback loop of RhoA–ROCK–Rnd3 has been shown to be involved in actin dynamics in membrane blebbing as well (Aoki et al., 2016). RhoE regulates the crosstalk between Rho/ROCK and RAF/MEK/ERK signalling pathways and is involved in FA and cytoskeletal organisation (Klein et al., 2008). The expression of RhoE is downregulated in rheumatoid arthritis and is transcriptionally regulated by FOXM1, which facilitates the inhibition of migration of fibroblast-like synovial cells through the Rho/ROCK pathway (Dai et al., 2022). Copper has been shown to promote the migration of mesenchymal bone cells by remodelling of the cytoskeleton by Rnd3 (Chen X. et al., 2020).

### 4.10 RhoBTBs

A very minimal amount of research has been done in elucidating the role of RhoBTB GTPases in cell migration and it is mostly centred around breast cancer wherein they primarily act as a tumour suppressor. RhoBTB1 has roles in maintaining Golgi morphology and cell invasion by altering the expression levels of the methyltransferase METTL7B (McKinnon and Mellor, 2017). Another study on MDA-MB-231 and MDA-MB-435 breast cancer cell lines, treated with ectopic expression of RhoBTB2, showed inhibition of the cells’ invasive properties (Ling et al., 2010). RhoBTB3 has been shown to similarly reduce cell invasiveness by reducing the expression of Col1a1 (Kim and Kim, 2022).

### 4.11 RhoH

A handful of studies done on RhoH with regards to migration show it as a regulator of Rac1. In an RNA interference screening of Rho signalling networks in prostate cancer, RhoH was found to be a regulator of Rac1, thereby involved in controlling cell migration (Tajadura-Ortega et al., 2018). The miR-3646/RhoH axis has roles in progression of coronary artery disease by augmenting several processes one of them being migration (Kang et al., 2018). RhoH deletion in hematopoietic progenitor cells led to chemotaxis and chemokinesis induced by an increase in stromal-derived factor-1alpha (SDF-1alpha). They also demonstrated that depletion of RhoH in cells increased Rac1 activity, as well as the colocalization of Rac1 with F-actin and lipid rafts at the cell membrane, and a significant enhancement in cell migration (Chae et al., 2008).

### 4.12 RhoF/Rif

RhoF has roles in filopodia formation, stress fibre formation and neuronal morphogenesis, all of which has been reviewed (Fan and Mellor, 2012). A constitutively active RhoF led to actin remodelling and an increase in single-cell migration (Shaverdashvili et al., 2019). In an extensive study on HCC, RhoF was found to be upregulated upon glucose deprivation. RhoF could upregulate glycolytic enzymes, causing a metabolic shift that resulted in the Warburg effect, which primarily mediated by RAB3D. They also demonstrated that HepG2 cells became spheroidal with fewer protrusions upon RhoF knockdown, and overexpression of the same led to a more mesenchymal morphology and larger, protrusive podosomes (Li S. et al., 2021). RhoF promotes EMT and migration in pancreatic cancer by upregulating c-Myc-mediated PKM2 transcription, which enhances glycolysis and drives lactate-induced Snail1 lactylation and nuclear translocation, thereby activating EMT (Zhao et al., 2024).

### 4.13 RhoD

RhoD is the only RhoGTPase expressed solely in mammals (Boureux et al., 2007). RhoD has roles in reorganizing actin filaments, dissolving stress fibres, and forming filopodia (Gad et al., 2012). The effects of RhoD knockdown and overexpression were shown to have different effects on the actin cytoskeleton and FA formation. For instance, overexpression of RhoD in HeLa, U2OS and U251MG cells led to formation of thin, intertwined actin filaments with reduced cortical actin (Blom et al., 2017). An increase in RhoD activity led to formation of filopodia containing actin as well, in HeLa and U251MG cells however not as much in U2OS cells (Blom et al., 2017). RhoD activated by FGF signalling can form filopodia like protrusions called cytonemes via mDia3C activation (Koizumi et al., 2012).

### 4.14 RhoU/Wrch1 and RhoV/Wrch2

In malignant plasma cells, RhoU is activated by the JAK/STAT signalling pathway, leading to cell migration (Canovas Nunes et al., 2018). Grb2 associates RhoU to EGFR signalling and cell migration (Zhang et al., 2011). RhoU also has roles in cranial neural crest cell migration and acts as a target for NOTCH and is involved in the migration of T-cell lymphoblastic leukaemia cells (Fort et al., 2011; Bhavsar et al., 2013). RhoU can be localized to adhesive structures and regulate the number and distribution, thereby increasing cell motility (Ory et al., 2007). Cdc42 effector protein PAK4 along with RhoU can aid in adhesion turnover and therefore cell migration (Dart et al., 2015). RhoV is involved in lung adenocarcinoma cell migration via LNK/c-Jun pathway (Zhang D. et al., 2021). A miR-1258/RHOV axis is involved in NSCLC metastasis (Wang R. et al., 2022). RhoV has also been found to be a pro-metastatic factor in TNBC (Jin et al., 2023).

## 5 Physiological and pathological implications of Rho GTPase signalling in cell migration

Rho GTPases, including RhoA, Rac1, and Cdc42, have been implicated in several pathological conditions, including cancer metastasis. The roles of Rho GTPases in cancer progression have been detailed in various reviews across the years, highlighting the signalling pathways involved in making them act as both oncogenes and tumour suppressors (Crosas-Molist et al., 2022; Clayton and Ridley, 2020; Liu et al., 2024; Svensmark and Brakebusch, 2019). Dysregulation of Rho GTPase signalling can contribute to the development and progression of cancer by altering various cellular processes, including cell proliferation, survival, migration, and invasion. Rac1 is a downstream effector of multiple signalling pathways, including PI3K/AKT, ERK, and EGFR. Its aberrant activation through overexpression of GEFs such as VAV3, PREX1, VAV2, and TIAM1 associates with various aggressive behaviours of breast cancer cells, including increased proliferation, invasion, survival, and resistance to therapy (Ji et al., 2022; Liu B. et al., 2021; Yamaguchi et al., 2020; Liang J. et al., 2021; Sosa et al., 2010; Zhang et al., 2022). The overexpression of Rac1 has been observed in various types of cancer, including breast cancer (BC), lung cancer, colorectal cancer, gastric cancer, prostate cancer, hepatocellular carcinoma, gallbladder cancer, and ovarian cancer (Liang J. et al., 2021; Du et al., 2012). A meta-analysis revealed that high Rac1 expression is associated with malignant phenotype, and a poor prognosis in cancer patients (Lou et al., 2018). Certain pathways lead to the spatial regulation of Cdc42, such as VEGF stimulation, which leads to the relocation of Cdc42 to the cell front of migrating CRC cells, resulting in enhanced metastasis through filopodia and invadopodia formation. This process can be clinically targeted to reduce CRC metastasis (Ma et al., 2020). Increased levels of RhoA are observed in different cancers to promote cell growth and metastasis. For example, hypoxia-induced RhoA upregulation drives an adaptive mechano-metabolic shift in gastric cancer by remodelling mitochondrial morphology via the ROCK pathway, ultimately synchronizing altered ROS production with enhanced actomyosin-mediated motility (Pal et al., 2025). Whereas, in some cancers decreased expression, deletion of RhoA resulting in low RhoA activity is shown owing to its tumour suppressor effect which suggests that RhoA function is either highly cell-type specific or inefficient cancer models (Svensmark and Brakebusch, 2019). Apart from wild-type RhoA various mutated forms are seen in cancers. For example, a gain of function mutation of RhoA is commonly found in diffuse gastric carcinoma that accounts for high malignant phenotype with increased infiltration (Kakiuchi et al., 2014).

As mentioned earlier, not only Rac, Rho, and Cdc42, but also other Rho GTPases, are overexpressed and affect the migration and metastasis of many cancers. The related pathways are potential therapeutic targets. Even pathways that are dysregulated due to the downregulation of certain Rho GTPases, which act as tumour suppressors, can be considered therapeutic targets. Apart from cancer, other pathological conditions can be treated by targeting pathways related to Rho GTPases. One such example is posterior capsular opacification (PCO), which occurs after cataract surgery and can lead to visual loss post-operatively. The migration causes this condition, as well as the presence of residual human lens epithelial cells, induced via FAK/Cdc42 signalling (Wen et al., 2022). RhoA being involved in various aspects of cell migration is often found to be dysregulated in various pathological states such cancer, neurodegenerative diseases, cardiac diseases *etc.* (Svensmark and Brakebusch, 2019; Guiler et al., 2021; Arrazola Sastre et al., 2020; Lin and Zheng, 2015; Santos et al., 2023).

Some of the potential strategies for targeting Rho GTPase signalling pathways can be A) Development of small molecule inhibitors that target Rho GTPase, B) Targeting upstream regulators of Rho GTPases, and C) Modulation of Rho GTPase activity in specific cells or tissues *etc.* Several preclinical studies have demonstrated the potential of Rac1 inhibitors as anti-cancer agents. For example, inhibiting Rac1 with EHop-016 and sorafenib combination therapy shows reducing tumour growth suggesting a promising potential therapeutic strategy for HCC (Ren et al., 2022). Moreover, GYS32661 and MBQ-167, a potent and selective inhibitor of Rac1, has been demonstrated in preclinical development for the treatment of advanced solid tumours (Bailly et al., 2022). However, Gene therapy or pharmacological induction of Rac1 activity also modulates Rho GTPase activity in specific cells or tissues, which can have therapeutic benefits. For example, overexpression of Rac1 in skin cells has been shown to promote wound healing by increasing cell migration and promoting the formation of new blood vessels. Mevastatin treatment-induced activation of Rac1 through EGF-mediated signalling can also promote keratinocyte migration and wound healing (Sawaya et al., 2019). Pharmacological inhibition by Fascin, a protein found in cellular protrusions in ovarian cancer and stromal cells led to a decrease in the migration of the both due to a decrease in the activities of Cdc42 and Rac1 (McGuire et al., 2019). Grinamycin B-F, a novel compound isolated from *Streptomyces* lusitanus, has been proven to exhibit antitumour efficacy by attenuating tumour sphere formation and decreasing tumour markers in glioblastoma multiforme (GBM) via alterations in the RhoA and PI3K3/AKT pathway (Yao Y. et al., 2020). Additionally, in Clear cell renal cell carcinoma (ccRCC), arctigenin a natural compound showed anti-migratory capability in a RhoA dependent manner which also exhibited synergistic action with existing drugs like 5-fluorouracil and sorafenib to prevent tumour growth in xenograft model (Liu D. et al., 2023). Eupafolin that is documented to have anticancer capabilities, inhibits cell migration, proliferation and invasion of non-small-cell lung cancer cells by downregulating RhoA and MMP9 expressions (Wang et al., 2023). Fluorometholone, a synthetic steroid supresses corneal epithelial hyperplasia by downregulating Rho GTPases—RhoA, Rac1, and Cdc42—and their downstream Erk and NF-κB pathways (Lin et al., 2025).

Research has also been conducted on natural compounds that act as metastatic inhibitors. For example, a natural compound called Cycloartocarpin, extracted from Moraceae (Artocarpus gomezianus), acts as an antimetastatic compound for lung cancer by downregulating Cdc42 expression, among other proteins, leading to a decrease in EMT (Tungsukruthai et al., 2022). Another natural flavonoid compound called Luteolin has been shown to inhibit Src/FAK and all the three Rho GTPases Rac1, Cdc42 and RhoA downstream of it and decrease cell migration and invasion in lung cancer (Masraksa et al., 2020). Combination treatments of Resveratrol (a polyphenolic plant antitoxin) and Dihydroartemisinin (a main active metabolite of anti-malarial drug artemisinin) have been shown to reduce migration in HepG2 liver cancer and MDA-MB-231 breast cancer cell line by acting on Cdc42 that in turn regulates JNK/NF-κB and N-WASP signalling pathways (Gao et al., 2020). Xihuang pill (XHP), a traditional remedy for breast cancer, suppresses tumour growth and metastasis by inhibiting epithelial–mesenchymal transition and promoting apoptosis. It modulates the AXL/TGF-β1/RhoA/ROCK and ERK signalling pathways leading to collective decrease in proliferation and migration of triple-negative breast cancer cells (Yang et al., 2025). The important targetable pathways related to Rac and Cdc42 in cancer treatment and the efficacies of inhibitors available have been discussed in a detailed review (Maldonado and Dharmawardhane, 2018). Apart from the Rho GTPases themselves and the signalling pathways, the RhoGEFs and RhoGAPs can also be targeted for impeding cancer metastasis which also has been discussed (Maldonado et al., 2020; Kreider-Letterman et al., 2022).

In conclusion, targeting Rho GTPase signalling pathways has the potential to be an effective therapeutic intervention for various diseases. Further research is needed to develop and optimize potential strategies for clinical use. However, it is important to note that the efficacy and safety of such interventions would need to be carefully evaluated through preclinical and clinical studies.

## Glossary

AP-1: Activator Protein 1
aPKCζ: Protein Kinase C Zeta
ARAP3: ArfGAP with RhoGAP domain, ankyrin repeat and PH domain 3
Arf: ADP-ribosylation factor
ARP2/3: Actin-related protein 2/3
Ascl1: Achaete-scute homologue
BCR: Breakpoint cluster region
C1P: Ceramide-1-phosphate
CCM: Collective cell migration
ccRCC: Clear cell renal cell carcinoma
Cdep: Chondrocyte-derived ezrin-like domain containing protein
CDR: Complementarity-determining regions
CerK: Ceramide kinase
CK1α: Casein kinase 1α
COUP-TFI: COUP Transcription Factor 1
COX-2: Cyclooxygenase-2
CRAI2: Calcium release-activated calcium modulator 2
CRC: Colorectal cancer
CTRP8: C1q/TNF‐related peptide 8
CXCR4: Cxcr4 chemokine (CXC motif) receptor 4
CYRI: CYFIP related Rac1 interactor
Daam1: Disheveled-associated activator of morphogenesis 1
DCIS: Ductal carcinoma in situ
DDR1b: Discoidin Domain Receptors 1b
DEPDC1B: DEP Domain Containing 1B
DLC1: Deleted in Liver Cancer 1
DOCK: Dedicator of cytokinesis
Dsh: Dishevelled
DUXAP8: Double homeobox A pseudogene 8
Ect2: Epithelial Cell Transforming 2
EGF: Epidermal growth factor
EGFR: Epidermal growth factor receptor
eIF3a: Eukaryotic translation initiation factor 3 subunit A
ELMO: Engulfment and cell motility protein 1
Eph: Ephrin
EPHA4: EPH Receptor A4
ERK: Extracellular signal-regulated kinase
ET-1: Endothelin-1
FA: Focal adhesion
FAK: Focal adhesion kinase
FARP1: FERM, ARH/RhoGEF and pleckstrin domain protein 1
FGD4: FYVE, RhoGEF and PH domain containing
FN: Fibronectin
FOXD3: Forkhead box D3
FYCO1: FYVE And Coiled-Coil Domain Autophagy Adaptor 1
Gab1: GRB2 Associated Binding Protein 1
GAPs: GTPase activating proteins
GAS5: Growth arrest-specific 5
GCN2: General control nonderepressible 2
GDIs: Guanine-nucleotide dissociation inhibitors
GEFs: Guanine nucleotide exchange factors
GIT1/2: G Protein-Coupled Receptor Kinase-Interactor 1/2
Gli1: Glioma-Associated Oncogene 1
GMPR: Guanosine Monophosphate Reductase
GPCR: G protein-coupled receptors
GRAF1: GTPase Regulator Associated with Focal Adhesion Kinase
Grb2: Growth Factor Receptor Bound Protein 2
HCC: Hepatocellular carcinoma
HIF1α: Hypoxia-inducible factor-1α
HMGA2: High Mobility Group AT-Hook 2
HSPC300: Haematopoietic stem/progenitor cell protein 300
IMPDH2: Inosine-5'-monophosphate dehydrogenase 2
IQGAP1: IQ-domain GTPase-activating protein 1
IRSp53: Insulin receptor substrate of 53 kDa
JAK3: Janus Kinase 3
LACTB: Serine Beta-Lactamase-Like Protein
LAPTM4B: Lysosome‐associated protein transmembrane 4B
LARG: Leukemia-associated RhoGEF
LIMK1: LIM kinase 1
LIMK2: LIM kinase 2
LMP1: Latent membrane protein 1
LOXL1-AS1: LOXL1 Antisense RNA 1
LPS: Lipopolysaccharides
LSD1: Lysine-specific demethylase 1
MAPK: Mitogen-activated protein kinase
MCAK: Mitotic centromere-associated kinesin
METTL7B: Methyltransferase Like 7B
Miro: Mitochondrial Rho GTPase 1
MKL1: Megakaryoblastic leukemia 1
MLC2: Myosin light chain-2
MRLC: Myosin regulatory light chain
MT: Microtubule
mTOR: Mammalian target of rapamycin
MYO1B: Myosin 1B
MYPT: Myosin phosphatase targeting subunit
NADPH: Nicotinamide adenine dinucleotide phosphate
NF-κB: Nuclear factor kappa-light-chain-enhancer of activated B cells
NHE1: Na+/H+ exchanger 1
NMII: Non-muscle myosin II
NOTCH: Neurogenic locus notch homolog protein
NSCLC: Non-small cell lung cancer
N-WASP: Neural- Wiskott-Aldrich Syndrome protein
OSCC: Oral squamous cell carcinoma
PAK: P21-activated kinases
Par3: Protease-activated receptors
PCO: Posterior capsular opacification
PDGF: Platelet-derived growth factor
p-ERK: Extracellular signal-regulated kinase
PI3K: Phosphoinositide 3-kinase
PIPKIγ: Phosphatidylinositol-4-phosphate 5-kinase gamma
PIX: p21-activated protein kinase exchange factor
β-PIX: p21-activated protein kinase exchange factor beta
PKC: Protein kinase C
PKCiota: Protein kinase Ciota
PKCα: Protein kinase Cα
PKL: Protein Kinase-Like
PLC-γ1: Phospholipase C-gamma1
PM: Plasma membrane
p-MEK: Mitogen-activated protein kinase kinase
PREX1: Phosphatidylinositol 3,4,5-trisphosphate (PIP3)-dependent Rac exchanger 1
PTEN: Phosphatase and TENsin homolog deleted on chromosome 10
PYCR1: Pyrroline-5-carboxylate reductase 1
Rab: Ras-like in brain
Rab5a: Ras-related protein Rab-5A
Rab6: Ras-related protein Rab-6A
RAF: Rapidly accelerated fibrosarcoma
Ran: Ras-like nuclear
Rap: Receptor-associated protein
Rheb: Ras homolog enriched in brain
Rho: Ras homology
Robo1: Roundabout Guidance Receptor 1
ROCK1: Rho-associated, coiled-coil-containing protein kinase 1
ROS: Reactive oxygen species
RP58/ZNF238: Zinc finger protein 238
RXFP1: Relaxin family peptide receptor 1
SACC: Salivary adenoid cystic carcinoma
Scar: Suppressor of cAR
SDF-1alpha: Stromal-derived factor-1alpha
SGEF: SH3-containing guanine nucleotide exchange factor
SOS1: Son of sevenless homolog 1
SRGAP1 SLIT-ROBO: Rho GTPase Activating Protein 1
StarD13: StAR-related lipid transfer domain protein 13
STAT3: Signal transducer and activator of transcription 3
TAZ: Tafazzin
TBX19: T-box transcription factor 19
TEAD: Transcriptional enhanced associate domain
TGFβ1: Transforming growth factor-β1
TIAM1: T-Lymphoma Invasion And Metastasis-Inducing Protein
TNBC: Triple negative breast cancer
TNF-α: Tumour necrosis factor alpha
TRIO: Trio Rho Guanine Nucleotide Exchange Factor
TRPC6: Transient Receptor Potential Cation Channel Subfamily C Member 6
TRPM7: Transient receptor potential melastatin 7
TRPV4: Transient receptor potential cation vanilloid 4
VASP: Vasodilator-stimulated phosphoprotein
VCA: Verprolin, cofilin, acidic
VEGF: Vascular endothelial growth factor
WASP: Wiskott-Aldrich Syndrome protein
WAVE: WASP-family verprolin-homologous protein
WHAMM: WASp homologue associated with actin Golgi membranes and microtubules
WRC: Wave regulatory complex
YAP: Yes-associated protein
ZFAS1: ZNFX1 Antisense RNA 1
